# Artificial “zombie” cells: An emerging platform for targeted drug delivery and disease treatment

**DOI:** 10.1016/j.apsb.2026.06.037

**Published:** 2026-06-29

**Authors:** Yu Sun, Jie Feng, Ye Xing, Ping Sun, Minghui Liao, Xiaoyan Tan, Yi Wang

**Affiliations:** a School of Life Science and Engineering, Southwest Jiaotong University, Chengdu 610031, China; b College of Medicine, Southwest Jiaotong University, Chengdu 610031, China; c Chongqing Three Gorges Medical College, Chongqing 404120, China; d Engineering Research Center for Biomimetic Synthesis of Natural Drugs, School of Life Science and Engineering, Southwest Jiaotong University, Chengdu 610031, China; e Sichuan Key Laboratory of Advanced Technologies of Materials Ministry of Education, School of Materials Science and Engineering, Southwest Jiaotong University, Chengdu 610031, China

**Keywords:** Artificial “zombie” cells, Targeted drug delivery, Cryogenic shock, Intracellular hydrogelation, Cryo-silicification, Cell fixation, Tumor vaccine, Inflammatory diseases

## Abstract

Living cell therapy represents a promising strategy for targeted drug delivery and personalized medicine. However, its clinical translation is limited by complex isolation procedures, stringent storage conditions, and inherent risks of pathogenicity. Derived from this approach, cell membrane-coated carrier technology also faces challenges related to scalability and intricate multistep fabrication. To overcome these limitations, artificial “zombie” cells (AZCs) therapy has emerged as an innovative alternative. This therapy employs simple methods, such as cryogenic shock, intracellular hydrogelation, or cryo-siliconization, to inactivate cells. This process eliminates potential pathogenicity while preserving membrane integrity and drug-loading functionality. Based on the intact cellular structure or engineering approaches, these “zombie” cells gain drug-loading capacity, targeting ability, and therapeutic potential. Compared to traditional therapies, AZCs therapy requires simplified processing and offers enhanced stability and improved safety, demonstrating promising efficacy in treating various cancerous and inflammatory diseases. Current AZCs exhibit diverse types, heterogeneous preparation methods and varied therapeutic mechanisms, highlighting the lack of systematic consolidation in this field. Therefore, this review comprehensively analyzes their preparation technologies, functionalization strategies, mechanisms of action, and disease applications of AZCs, to evaluate current research progress and clinical potential, offering insights to advance preclinical-to-clinical translation.

## Graphical abstract

AZCs: An emerging platform for targeted drug delivery and disease treatment.

**Figure:**
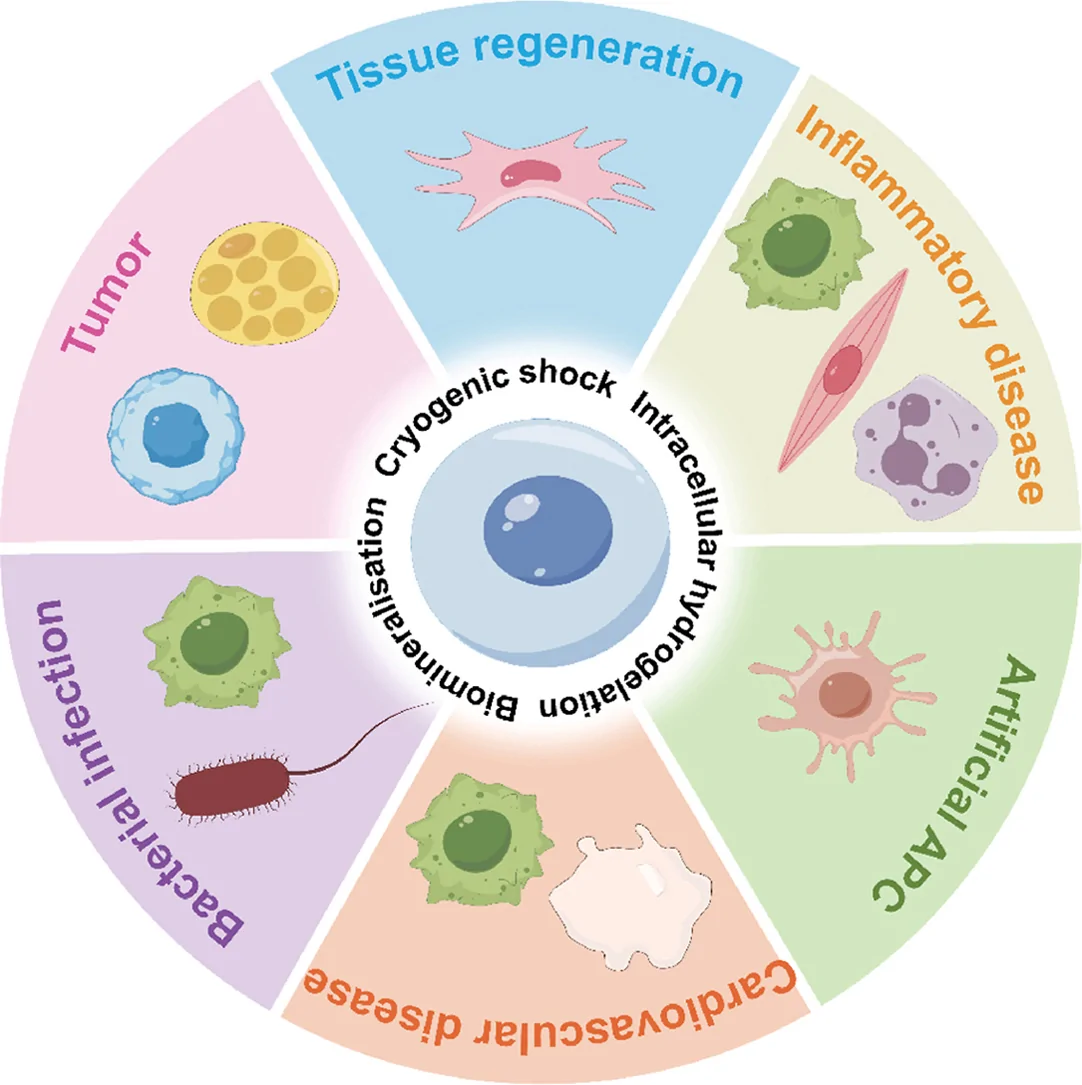

## 1 Introduction

Living cells serve as promising carrier materials due to their innate targeting capabilities and achieve multi-organ localization and transport with proteins on their membrane^1^. Meanwhile, they exhibit prolonged circulation half-life, low blood and tissue clearance rates, and minimal biotoxicity^2^^,^^3^. However, their inherent immunogenicity may trigger host immune responses and lead to carrier clearance. Additionally, metabolic interference between living cells and therapeutic drugs may reduce drug efficacy and compromise treatment outcomes^4^^,^^5^. Moreover, significant challenges arise from the instability of living-cell drug carriers, poor storage stability, requirement for fresh preparation, and lack of stock for emergency use^6^^,^^7^. Fortunately, these limitations are addressed by cell membrane coating nanotechnology^8^, a derivative of living-cell delivery systems. While maintaining the inherent tissue-targeting capability, cytokine/antibody clearance, and toxin neutralization properties of natural cells, this technology simultaneously overcomes the immune response triggered by intact living cells^9^^,^^10^. However, the extraction of cell membranes is complex and causes disruption of their native structure^11^^,^^12^, which frequently induces denaturation of surface proteins^13^ and compromises carrier functionality. Furthermore, the process suffers from low yield, demands stringent storage conditions, and yields nanoparticles which hardly meet practical requirements. These challenges collectively hinder clinical translation^14^^,^^15^.

In that context, to preserve cell membrane protein targeting capabilities and streamline preparation, researchers developed the AZCs strategy^16^, widely applied in treating various diseases nowadays. Techniques including cryogenic shock^17^, intracellular hydrogelation^18^, and biomineralization^19^ render cells inactive, eliminate pathogenicity while maximizing preservation of cellular integrity and membrane functionality^20^. These approaches prevent efficacy reduction from living-cell pathogenicity and metabolic reactions while resolving targeting deficiencies caused by structural disruption and, enable large-scale production^21^. In summary, AZCs technology masterfully integrates the innate targeting capacity of natural cells with the tunable properties of engineered materials, establishing a unique therapeutic platform. Its key strengths lie in the convergence of programmable functionality and biosafety—by loading diverse therapeutic agents or engineering surface modifications, their therapeutic actions can be precisely tailored. Furthermore, the inactivated cellular carriers eliminate risks associated with living cells, such as tumorigenicity and immune rejection. Together, these attributes position AZCs as a pivotal technological foundation for advancing the next generation of precise, potent, and safe cell-based therapies.

The diversity of natural cells enables generation of varied AZCs. Diverse preparation and engineering techniques modify cell constructs to enhance tissue tropism, cell adhesion, and cytokine clearance, thereby expanding AZCs diversity. Considering this diversity, a systematic summary for these variants is needed when research about how preparation and engineering methods affect their functions remains limited. A comprehensive overview of living cell-based therapeutics was provided by Yang et al.^3^, however, the discussion of inactivated cell systems remained relatively peripheral. Similarly, the broader review by Kuang et al.^22^ on bio-organism-inspired drug delivery platforms, while including a subsection on “zombie” cells, fell short of providing a systematic and in-depth methodological examination. Similarly, the applications of “zombie” cells prepared *via* liquid nitrogen cryo-shock in cancer and inflammation have been reviewed by Wang et al.^23^ However, their investigation was limited in scope, focusing solely on a technique utilizing liquid nitrogen cryogenic shock for preparing “zombie” cells. In contrast, our work presents the first comprehensive summary of all currently available methods for preparing AZCs, provides detailed explanations of the underlying principles for each technique, and offers a systematic multi-dimensional comparison to assist readers in selecting the most appropriate preparation method with greater clarity and precision. Building upon this foundational understanding, this review therefore comprehensively concluded advances in AZCs for biomedical applications and defines AZCs and their key characteristics for the first time. Secondly, the applications and differences in the use of AZCs prepared by different techniques in diseases such as tumors, tumor vaccine, immunotherapy and systemic inflammatory response syndrome were reviewed. Finally, the advantages and challenges of AZCs as biomimetic vectors were presented, and the prospects for future applications were outlined.

## 2 Artificial “zombie” cells

### 2.1 Definition of AZCs

Senescent cells represent a naturally arising category of “zombie” cells, characterized primarily by permanent cell cycle arrest coupled with a dynamically maintained senescence-associated secretory phenotype (SASP). Although these cells lose their proliferative capacity, they remain metabolically active. Through the secretion of various inflammatory factors, proteases, chemokines, and other signaling molecules, they can inflict sustained damage on the surrounding tissue microenvironment, thereby promoting organismal aging and the progression of related diseases^24^. It is worth noting that in biological contexts, the term “zombie” cells are sometimes used interchangeably with senescent cells. However, a critical distinction exists in their underlying mechanisms and implications: naturally arising “zombie” cells specifically refer to senescent cells, which enter an irreversible growth arrest in response to stress or damage *in vivo*, while retaining metabolic activity and SASP effects. In contrast, the AZCs referred in this study are artificially engineered cellular “corpses” designed to preserve structural integrity without cellular activity. Accordingly, to facilitate clarity and distinction, this review will consistently use “senescent cells” to denote the naturally occurring entities and “AZCs” to refer to the experimentally fabricated cellular constructs^25^. Senescent cell clearance strategies aim to selectively induce apoptosis in senescent cells or modulate their SASP to mitigate pathological effects, representing a “clearance-based” therapeutic approach. In contrast, AZC strategies utilize their stable, inactive cellular structures as functional materials in fields such as tissue engineering, immune regulation, and drug delivery. These two approaches differ fundamentally in their formation mechanisms, biological characteristics, and applications: senescent cells emerge naturally in response to physiological stress or damage, exhibiting distinct SASP activity and microenvironment-disrupting capacity^26^. While AZCs are lifeless constructs formed through exogenous intervention, they lack secretory capacity or direct damaging effects, which makes them better suited as biocompatible materials or tools for microenvironment modulation.

In contrast, AZCs are cell corpses fabricated through diverse physical and chemical processes that preserve cellular structure, eliminate cell viability and proliferative capacity 27, 28, 29. These artificially engineered “zombie” cells preserve the mechanical properties of native cell membranes and retain the ability to bind signaling molecules, enabling applications in targeted drug delivery, immunotherapy, and clearance of cytokines or toxins. According to the basic characteristics of AZCs, we believe the defining characteristics of AZCs should comprise: (1) cellular corpses with complete membrane structures generated by physical or chemical methods; (2) tissue/lesion chemotaxis capabilities and membrane-mediated interactions with living cells or endogenous factors; and (3) additional therapeutic regulatory functions associated with cell structure-based drug delivery.

### 2.2 Biological properties of AZCs

The AZCs produced with aforementioned methods maintain the original morphological structure. It is worthwhile noting that the glass transition, hydrogel network, or mineralized structure collaboratively stabilize the cell membrane and internal ultrastructure. On that note, the benefits of AZCs lie in their distinctive ability to preserve the cell membrane structure intact while halting metabolism. This feature provides natural advantages in targeted therapy and immune regulation applications.

AZCs exhibit notable distinctions from living cells in metabolic activity, structural dynamics, functional responsiveness, and molecular regulatory mechanisms^30^^,^^31^. Living cells uphold regular physiological functions through the balance between catabolic and anabolic processes, efficient energy conversion coupling, and a multi-tiered regulatory network^32^. On the contrary, the loss of mitochondrial function in AZCs leads to metabolic arrest, thereby impairing their ability to maintain basic cellular functions^33^. Living cells dynamically respond to external stimuli, whereas AZCs show minimal or no detectable response^34^. The molecular regulatory system in living cells is a complex, integrated network that includes gene expression regulation, protein modification, signal transduction pathways, metabolic balance, and cell cycle control^35^^,^^36^. These systems work together to maintain cellular homeostasis through precise coordination, allowing cells to respond to external stimuli^37^. In AZCs, essential metabolic processes are disrupted, leading to the inactivation of key biological molecules like enzymes and signaling proteins. As a result, AZCs lose the ability for molecular regulation, which is crucial for the consistent therapeutic efficacy of AZCs^38^^,^^39^. Living cells achieve and sustain homeostasis through processes such as mitosis and autophagy, which can be likened to cell “production” and “cleaning” mechanisms^40^. Conversely, as non-living entities, AZCs, exhibit a relatively static state compared to the dynamic motion observed in living cells^41^. The contrasting biological behaviors of AZCs and living cells can be attributed to the deliberate disconnection between metabolic termination and structural preservation in the former. While AZCs maintain a fixed ultrastructure by sacrificing dynamic functions, living cells rely on continuous metabolism to support vital activities.

### 2.3 Preparation of AZCs

#### 2.3.1 Cryogenic shock

**Figure 1:**
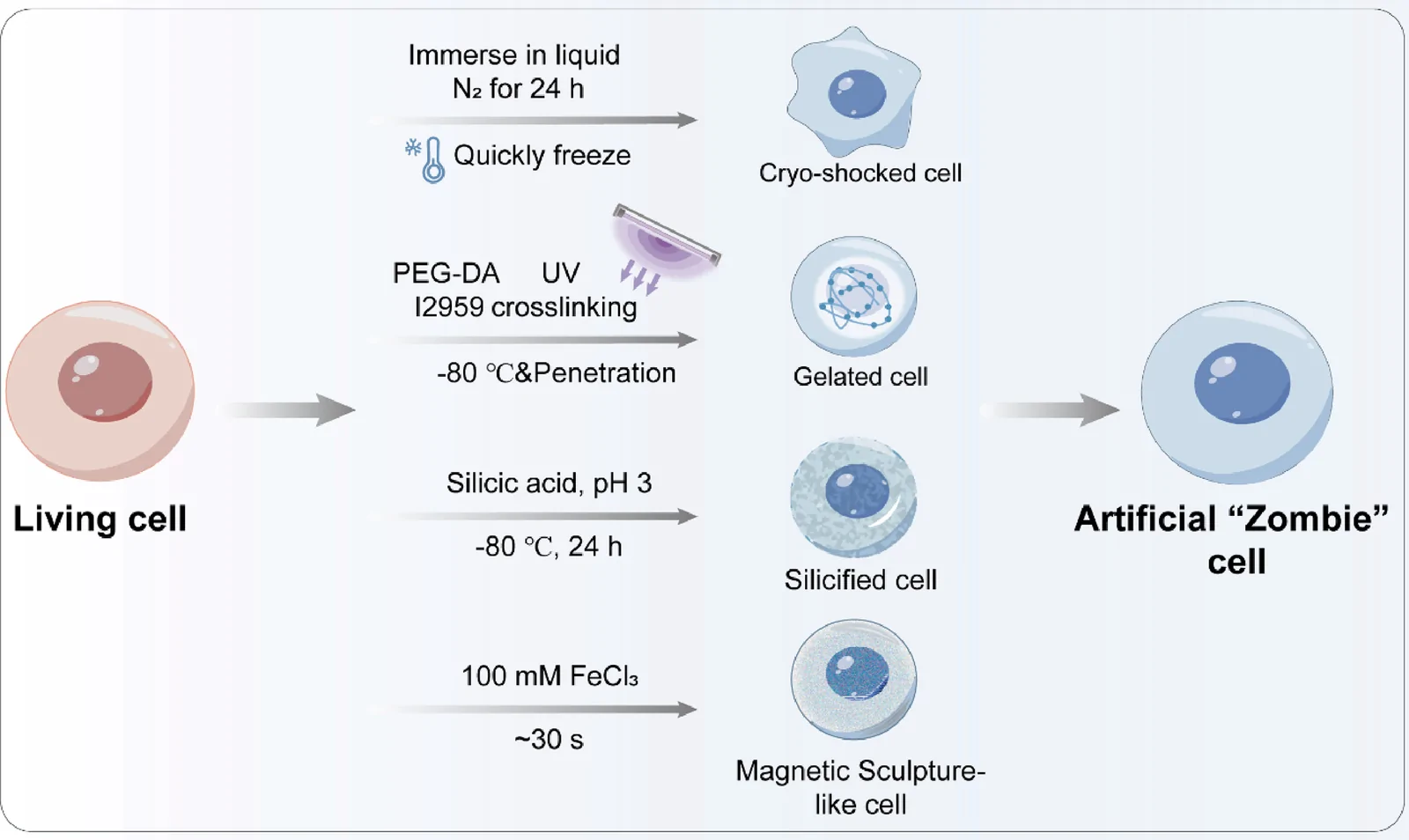
Schematic representation of AZCs preparation *via* distinct methodologies: cryo-shocked cells are obtained by subjecting living cells to rapid cryo-treatment with liquid nitrogen; hydrogel precursor molecules and a photoinitiator enter living cells through autonomous permeation or freeze-thaw methods, and are crosslinked into a hydrogel network *via* ultraviolet irradiation to produce gelated cells; living cells were biomineralized in silicate solution at an ambient temperature of −80 °C for 24 h to obtain silicified cells; magnetic sculpture-like cells are generated by treating live cells with high-concentration FeCl_3_ for 30 s.

Cryogenic shock technology achieves simultaneous glass transition of intracellular and extracellular water *via* rapid liquid nitrogen cooling under the protection of cell cryopreservation medium, thereby preventing structural damage induced from ice crystal formation^42^^,^^43^ (Fig. 1). During this process, the cytoplasm and extracellular matrix undergo the formation of an amorphous solid glass phase at −196 °C. This vitrification enables *in situ* fixation of three-dimensional cellular architecture, including microfilament-microtubule networks and membrane protein distributions caused by inhibiting intracellular phase segregation and biomacromolecule denaturation^22^. Since cells undergo rapid vitrification through direct exposure to liquid nitrogen rather than controlled cooling to preserve ultrastructure, the mitochondrial metabolism, enzyme activity, and cell division will be terminated^44^. Consequently, cells progress to late-stage apoptosis due to subcellular dysfunction^45^.

#### 2.3.2 Intracellular hydrogelation

Intracellular hydrogelation entails incorporating hydrogel materials and polymer precursor molecules into living cells. Subsequent photoactivated crosslinking within the cytoplasm forms stable hydrogel networks that immobilize cytoskeletal and subcellular structures^46^ (Fig. 1). The hydrogel network formation not only immobilizes cellular structures but also impedes essential metabolic processes, leading to mitochondrial dysfunction, cessation of metabolic activities, and ultimately, cellular inactivity^47^. Compared to cryogenic shock-generated cellular constructs, those cells produced *via* intracellular hydrogelation possess a more robust internal hydrogel structure, which enhances cellular stability and may improve controlled drug slow release capacity^48^, and more importantly, preserves the membrane lipid fluidity, lipid order, and protein mobility. Owing to their inherently biomimetic architecture with organized and fluid membrane structure these AZCs show significant advantages in biomedical applications antigen-presenting cells (APCs)^18^, cell decoys^48^ and cell vaccines^49^.

#### 2.3.3 Biomineralization

Except for conventional cryogenic shock and intracellular hydrogelation discussed above, AZCs can also be produced *via* techniques such as freeze-induced siliconization and FeCl_3_ chemical induction (Fig. 1). Cryo-silicification diffuses silica precursors into cryopreserved cells and nuclei, followed by hydrolytic polycondensation catalyzed by intracellular enzymes. This process employs freezing at −80 °C to slow silicic acid condensation kinetics while inhibiting enzymatic activity to prevent autolysis. Concurrently, it promotes silica deposition throughout cellular compartments so as to achieve structural fixation^50^^,^^51^. Silica formed by silicic acid condensation stabilizes lipid and protein conformations in cell membranes, facilitating mineralization and structural fixation^52^. On the other hand, FeCl_3_ induces cross-linking reactions through Fe^3+^ binding to phospholipids, proteins, and intracellular anionic sites, generating a rigid polymeric network. The hypertonic environment created by a high concentration of FeCl_3_ leads to cell dehydration and accelerates cell shrinkage^53^. Elevated FeCl_3_ concentrations prompt metal coordination cross-linking within cells, initiating physical mineralization to fortify cell structures and create cell formations resembling sculptures^54^. The mineral fixation process represents a form of cell demise that is distinct from biological mechanisms, relying instead on physicochemical structural alterations^55^. The membrane of the “zombie” cell is clearly distinguishable from the nuclear membrane, and the presence of a cytoplasm and a granular nucleus is evident. These features are attributed to metal coordination cross-linking involving Fe^3+^ and intracellular structures^56^. These two methods have been shown to be effective in preserving the complete structure of the cell, thereby ensuring the maintenance of essential bioactive components, including cell membrane receptors and surface antigens. Furthermore, these cells are capable of engineering properties that surpass those of natural cells, thus acquiring the characteristics of what are termed AZCs.

These approaches for fabricating AZCs transcend conventional biological constraints, converting cells from “living organisms” into functional programmable cell biomaterials, which offers quantifiable and standardized biological instruments for targeted delivery, tumor treatment, cancer vaccines development, and related domains^57^^,^^58^, underscoring its promising clinical translational prospects. The above methods of preparing AZCs were analyzed and the technical profiles, methods characterization and advantages of AZCs prepared by each method were summarized (Table 1).

**Table 1:** Characteristics of AZCs preparation methods.

Approach	Technical profile	Characterization	Drug loading capacity	Deployed cell type	Advantages	Ref.
Cryogenic shock	Materials: Noncontrolled-rate cell cryopreservation medium Cost: Moderate Apparatus: Liquid nitrogen storage devices Preparation time:12‒24 h	SEM Live-dead staining Apoptosis flow cytometry Evaluation of CCK-8 proliferative activity	High, current resear-ch encompasses both intracellular and extracellular drug loading strategies.	Tumor cells, adipocytes, M1-like macrophage, neutrophils, platelet, mesenchymal stem cells, macrophages	1. Simple and rapid procedure, enabling standardized protocols. 2. Highly preserved surface antigens and homing capability of source cells. 3. Low immunogenicity with inherent biodegradability.	16 26 27 60 64
Intracellular hydrogelation	Materials: Poly (ethylene glycol) diacrylate/I2959 Cost: Moderate Apparatus: Ultraviolet light Preparation time: 10 min	TEM Fluorescence recovery after photobleaching Laurdan dye staining Total internal reflection fluorescence Western blotting	Relatively high, current research encompasses both intracellular and extracellular drug loading strategies.	Tumor cells, macrophages, dendritic cells, cardiomyocytes	1. High-efficiency preservation of source cell surface antigens and homing capacity. 2. Hydrogel internal structure enabling long-term, sustained drug release. 3. High stability, allowing for long-term room temperature storage after freeze-drying.	18 21 46 49 111
Cryo-silicification	Materials: Silicic acid Cost: Moderate Apparatus: Ultra-low temperature refrigerator Preparation time: 24 h	SEM Energy dispersive X-ray analysis Live-dead staining	Current research is limited to the extracellular loading of immune adjuvants.	Tumor cells	1. High-efficiency preservation of source cell surface antigens. 2. Highly functionalizable membrane surface. 3. Stability suitable for long-term storage.	19 52 53 54
FeCl_3_ chemical induction	Materials: FeCl_3_ Cost: Low Apparatus: — Preparation time: 30 s	SEM Proteomic analysis Live-dead staining Apoptosis flow cytometry Evaluation of CCK-8 proliferative activity	The current study does not encompass drug delivery.	Tumor cells	1. Rapid cell immobilization with low cost, facilitating a standardized preparation process. 2. Long-term storage stability. 3. High-efficiency preservation of source cell surface antigens.	38 39 40

### 2.4 AZCs versus cell-based carriers

Living cells, cell membrane-coated carriers, and AZCs can be viewed as distinct developmental landmarks in biomaterial-based therapeutic strategies, with each offering a unique set of advantages and limitations. Living cell therapies, with their intact biological activity, are capable of performing sophisticated functions such as active chemotaxis and complex signaling responses. However, their clinical translation is mainly constrained by unpredictable *in vivo* behavior, potential safety risks, and complex manufacturing processes. Cell membrane-coated carrier technology, which combines natural cell membranes with synthetic nanoparticle cores, ingeniously retains the source cell's surface recognition and immune evasion capabilities while circumventing the proliferation risks associated with living cells. Nevertheless, their functionality is largely confined to membrane protein-mediated interactions, and challenges remain regarding the structural stability of the carriers and drug encapsulation efficiency. In contrast, AZCs aim to integrate the advantages of both predecessors. By preserving the intact cytoskeleton and membrane structure, AZCs not only retain the inherent chemotactic and interactive abilities of living cells but also ensure high biosafety and controllability due to their non-living nature. What's more, their stable structure offers the potential for high-efficiency drug loading, and the preparation process is more amenable to standardization. Therefore, AZCs demonstrate considerable potential to outshine existing platforms, as they two core bottlenecks must be addressed: the unpredictability of living cell therapies and the functional limitation of membrane-coated carriers. Furthermore, through engineered biomimetic design and precise modulation, AZCs hold promise in overcoming critical challenges in the fields of drug delivery and cell therapy. A comparative summary of living cells, cell membrane-coated carriers, and AZCs as drug delivery vehicles across multiple dimensions is presented in Table 2.

**Table 2:** Comparisons of living cell, cell membrane-coated carrier, and AZCs.

Comparison dimension	Living cell	Cell membrane-coated carrier	AZCs
Core structure and composition	Intact living cells	A synthetic nanoparticle core coated with a natural cell membrane.	Cell corpses with intact membrane and nuclear structures
Key biological functions	Environmental sensing, active chemotaxis, and execution of complex biological reactions ^30^.	Inherits the targeting and immune evasion functions of the membrane surface.	Inherits chemotactic and interactive capabilities, coupled with additional therapeutic regulatory functions associated with cellular structure.
Cargo loading capacity	Low, highly dependent on cellular vitality.	Low encapsulation efficiency, unstable carrier structure, and prone to premature leakage ^110^.	High, the stable structure enables high loading with minimal risk of drug leakage ^16^.
Preparation and standardization	Significant batch-to-batch variation and a complex cell culture environment	The sophisticated manufacturing process hinders robust inter-batch standardization.	The preparation process is straightforward and readily amenable to standardized production ^27^.
Safety	Risks include uncontrolled proliferation, differentiation, and immune rejection; *in vivo* behavior is difficult to predict ^4^.	Relatively high level of safety. The core is composed of synthetic materials and poses no risk of proliferation ^11^.	Relatively high level of safety; non-proliferative, and highly biocompatible ^66^.
Stability *in vivo*	Due to their inherent viability, the *in vivo* stability of cells is unpredictable ^34^.	Due to the influence of core materials and membrane stability, a certain cycle time is typically required ^105^.	Can be engineered for either long-term circulation or on-demand degradation through material design.
Key technological challenges	Safety, delivery efficiency	Preserving membrane functionality and ensuring structural stability	Standardized preparation

**Table 3:** Examples of artificial “zombie” cell-based drug delivery carriers organized by cell type.

Cell type	Engineering method	Payload	Loading strategy & method	Applications in disease treatment	Ref.
AML cells	Cryogenic shock	Doxorubicin	Intracellular & cellular uptake	Increase the targeted delivery of doxorubicin in the bone marrow. Enhance the anti-tumor immune response.	16
B16F10 cells	Cryogenic shock	Imiquimod -loaded maleimide-liposomes	Extracellular & click chemistry	Achieve efficient co-uptake of antigens/adjuvants by antigen-presenting cells and prolong the *in-vivo* retention of tumor antigens and adjuvants.	101
	Cryogenic shock	Doxorubicin	Intracellular & cellular uptake	Target delivery of drugs and enhance the anti-tumor immune response.	118
4T1 cells	Cryogenic shock	Adamantane modified nanoparticles	Extracellular & host-guest interaction	Enhance the anti-tumor immune response and effectively inhibit distant tumors and lung metastases.	123
	Cryogenic shock	Sonosensitizer-loaded liposomes	Extracellular & click chemistry	Precisely deliver sonosensitizers to enhance anti-tumor efficiency and safety.	77
	Cryogenic shock	–	–	Cryo-shocked cancer cell microgels enhance the anti-tumor immune response, significantly reducing the tumor recurrence rate and metastasis efficiency.	137
Lewis lung carcinoma cells	Cryogenic shock	10-Hydroxycamptothecin and Quillaja saponin-21	Extracellular & physical interaction and chemical conjugation	Targeted pulmonary drug delivery to enhance the anti-tumor immune response.	130
A549 cells	Cryogenic shock	Lipofectamine 3000–pCas9/gCDK4 nanoparticles	Extracellular & electrostatic interactions	Targeted pulmonary drug delivery to increase the accumulation of drugs in the lungs.	66
TC-1 cells	Cryogenic shock	Oncolytic adenovirus type 11	Intracellular & cellular uptake	Targeted pulmonary adenovirus delivery to increase the accumulation of adenovirus in the lungs.	67
U251 cells	Cryogenic shock	Oncolytic adenovirus type 5	Intracellular & cellular uptake	Continuously release TAAs and oncolytic adenovirus, induce tumor lysis, and enhance the anti-tumor immune response.	120
Adipocytes	Cryogenic shock	Triptolide palmitate and photosensitizer Ce6	Intracellular & cellular uptake	Targeted pulmonary drug delivery effectively inhibits the proliferation and invasion of lung metastatic melanoma cells.	129
M1-like macrophage	Cryogenic shock	–	–	As a TLR2 agonist, it induces persistent M1 phenotype polarization both *in vitro* and *in vivo* and amplifies the expression of CD47 in tumor cells.	94
Neutrophils	Cryogenic shock	–	–	Neutralize various pro-inflammatory cytokines and endotoxins to improve the survival rate of septic mice.	27
	Cryogenic shock	Platinum nanoclusters	Extracellular & Biotin-streptavidin-biotin binding	Platinum nanoclusters are delivered to target brain and inflammation sites to decompose free radicals and reduce oxidative damage in mouse brain tissue.	168
	Cryogenic shock	Bacterial magnetosomes	Extracellular & Zr (IV)-phosphate bridge	Targeted delivery of bacterial magnetosomes to premetastatic niche sites to deplete tumor-derived secretory factors and inhibit tumor metastasis.	177
Platelet	Cryogenic shock	Liposomes loaded with thrombolytic drugs and anti-inflammatory drugs	Extracellular & host-guest interaction	Deliver drugs to target the thrombus site and increase the drug accumulation at the thrombus site.	170
Mesenchymal stem cells	Cryogenic shock	–	–	Accelerate skin wound healing.	174
Macrophages	Cryogenic shock	Atorvastatin-loaded nanoparticles	Intracellular & cellular uptake	Targeted plaque drug delivery to enhance macrophage immunity.	162
	Cryogenic shock	Quercetin-loaded nanoparticles	Extracellular & host-guest interaction	Targeted delivery of drugs to the site of pulmonary inflammation effectively alleviates acute pneumonia.	64
	Cryogenic shock	Shikonin	Intracellular & cellular uptake	Trigger efferocytosis in M1 macrophages and neutralize inflammatory cytokines.	60
	Intracellular hydrogelation	–	–	Effectively neutralize multi-target inflammatory cytokines and inhibit the progression of arthritis.	48
	Intracellular hydrogelation	Nissle1917	Extracellular & receptor-ligand interaction	Target colitis tissues, prolong the intestinal retention time of Nissle 1917, and neutralize pro-inflammatory cytokines.	84
	Intracellular hydrogelation	Superparamagnetic iron oxide nanoparticles	Intracellular & cellular uptake	Effectively capture and detect different pathogens, and also be able to respond to external magnetic forces to collect pathogens.	68
Dendritic cells	Intracellular hydrogelation	–	–	Induce an extended expansion time of mouse T cells and enhance the anti-cancer efficacy of adoptive T cell therapy.	21
Cardiomyocytes	Intracellular hydrogelation	–	–	Capture and neutralize coxsackievirus B3 through receptor‒ligand interactions, effectively inhibiting CVB3-induced viral myocarditis.	111
2F7-BR44 cells	Cryo-silicification	TLR agonists	Extracellular & electrostatic interactions	Delivering whole tumor antigen to activate DCs, enhancing T-cell response with rituximab, establishing long-term immune memory and inhibiting tumor recurrence.	57
PLC-PRF-5 cells	FeCl_3_ chemical induction	–	–	Promotes dendritic cell maturation and T-cell responses to tumors, and effectively inhibits melanoma growth in combination with adjuvants.	54

### 2.5 Potential risks of AZCs

Although AZCs have demonstrated high safety, excellent biocompatibility, and broad applicability in targeted delivery, tumor therapy, and cancer vaccine development, they still present several potential risks that vary in terms of the cell type and therapeutic purpose (Table 3). Acute myeloid leukemia (AML) cells treated with liquid nitrogen (referred to as LNT cells) were developed, and their potent efficacy was also demonstrated in a murine leukemia model. While the authors emphasized the importance of addressing cell proliferation—as residual proliferative activity in tumor-derived cells could lead to tumor formation—and confirmed that their LNTs lacked such activity, the methods and materials used in their study were relatively limited^16^. Consequently, the conclusions drawn may not be universally applicable. Therefore, when preparing AZCs from tumor cell sources, evaluating proliferative capacity must be considered a top priority. Meanwhile, current research on artificial “zombie” cancer cells lacks evaluation regarding long-term safety, making it impossible to determine whether these AZCs could lead to subsequent cancer after an extended period following treatment completion (greater than one year or more). For example, AZCs derived from M1 macrophages were used for the treatment of acute lung injury. While the administration of untreated M1 macrophages alone elicited significant inflammatory stimulation—attributable to their secretion of pro-inflammatory factors—the AZCs formulation eliminated this secretory capacity^28^. Nonetheless, the study revealed the retained immunostimulatory potential of such engineered cells. This consideration becomes particularly indispensable when selected parent cells possess intrinsic immunogenicity. For instance, dendritic cells (DCs) were utilized as the source material for AZCs. As DCs naturally express high levels of immune-activating surface markers such as CD80, CD86, and MHC II following induction, they inherently exhibit strong immunostimulatory properties^18^. While this characteristic offers advantages in certain therapeutic contexts, the retention of membrane proteins or genetic material during AZCs preparation implies these proteins may still exert antigenic functions, potentially triggering unintended immune responses *in vivo*. In some cases, such responses may take a toll on the intended therapeutic outcome. Consequently, the selection of cell sources for AZCs-based therapies demands comprehensive and prudent evaluation, alongside rigorous assessment of their potential immunogenicity.

## 3 Core functional properties of AZCs

### 3.1 Self-targeting ability

AZCs possess an intact cellular structure and membrane proteins that provide them with endogenous targeting capabilities^59^^,^^60^. Despite the development of numerous nano-pharmaceuticals in recent researches to enhance drug specificity and reduce side effects^61^, studies indicate that only a tiny fraction (approximately 0.0014%) of these nano-pharmaceuticals could reach cancer cells, even when nanoparticles are equipped with cancer cell recognition ligands. This significantly limits the therapeutic efficacy of drugs^62^. In the initial investigations utilizing cryogenic shock technology to create AZCs, researchers discovered that AML cells possess a natural ability to home to the bone marrow due to the presence of chemokine receptors CXCR4 and CD44^63^. Based on this trait, AML cells can serve as effective carriers to transport doxorubicin, a crucial chemotherapy drug for AML, to bone marrow lesions for great therapeutic outcomes. Subsequent experiments validated that LNT cells maintained the integrity of CXCR4 and CD44 receptors, thereby significantly enhancing the targeted delivery efficiency of doxorubicin within the bone marrow microenvironment. AML cells treated with cryogenic shock in advance eliminated the tumorigenicity of tumor cells and the drug loading per 1 × 10^7^ LNT cell reached 65 ± 16 μg. The precise preservation of intact cell membranes in AZCs—with their functional receptors remaining largely undisturbed—provides a unique platform for targeted drug delivery through receptor-ligand interactions or strategic molecular modifications^16^.

AZCs leverage their preserved surface protein characteristics and size to achieve both active and passive targeting toward pathological sites, a capability generally missing in cell membrane-coated carriers. Specifically, the core of cell membrane-coated carriers typically consists of nanoparticles, and the final membrane-coated structures usually measure only 100–200 nm in diameter. This size inevitably facilitates recognition and clearance by the reticuloendothelial system^9^. Furthermore, the fabrication process for cell membrane-based nanocarriers is complex, costly, and involves numerous critical considerations. The essential step of cell lysis to isolate membranes often induces conformational changes in surface proteins, leading to functional loss^11^. In contrast, AZCs technology effectively circumvents these issues. Current methods for preparing AZCs do not compromise membrane integrity, which largely preserves the function of membrane-associated proteins and significantly enhances the stability of AZCs. Additionally, the preparation of AZCs is typically completed in one or two steps. This straightforward process considerably reduces production costs and facilitates standardized manufacturing and clinical translation. For instance, in a representative application, an artificial “zombie” macrophage platform was successfully developed by utilizing the inherent chemotactic properties of living macrophages, thereby preserving the inflammatory tropism^64^.

Through cryogenic shock technology, they successfully retained the complete surface receptor profile of donor cells and created a stable drug delivery vehicle with inherently inflammatory targeting capability. In murine models of acute pneumonia, these bioengineered “zombie” macrophages effectively delivered nanoparticles to inflamed pulmonary tissues *via* endogenous chemotactic hitchhiking mechanisms. This strategy significantly mitigated pathological inflammation markers and ameliorated disease progression in treated subjects^65^. In addition, a lung-targeted CRISPR-Cas9 drug delivery strategy based on passive and active dual-targeting was proposed, in which they utilized liquid nitrogen cryo-shock treatment of lung cancer cells to obtain artificial “zombie” cancer cells that retained the intact cellular structure and the cell-surface glycoprotein CD44, which could achieve highly targeted lung delivery through passive capture of lung capillaries, CD44-mediated interaction and adhesion to lung endothelial cells. The artificial “zombie” cancer cells retained the intact cell structure and the cell surface glycoprotein CD44, and this cell carrier was able to realize highly targeted lung delivery through passive capture by lung capillaries and CD44-mediated interactions and adhesion of lung endothelial cells^66^. AZCs can achieve precise delivery and efficient enrichment in lesions by integrating the dual strategies of active and passive targeting. This synergistic mechanism not only markedly improves the bioavailability of therapeutic drugs, but also reduces the systemic toxicity, providing an innovative solution to overcome the off-target effect of traditional nanomedicines.

### 3.2 Cargo loading ability

#### 3.2.1 Intracellular loading

AZCs primarily serve as drug delivery vehicles through two loading methods: intracellular and extracellular loading. Intracellular loading utilizes the phagocytic capacity of cells to internalize therapeutic agents or nano-delivery systems, followed by cryogenic shock treatment and intracellular gelation techniques to construct biomimetic drug delivery platforms. Common cargo types for intracellular loading include small molecule drugs, nanoparticles encapsulating small molecules, and viruses. While small molecules can penetrate cell membranes, they are prone to leak. Consequently, encapsulating small molecules within nanoparticles not only extends their systemic circulation time but also significantly enhances drug loading capacity. The successful loading of nanoparticles or viruses relies fundamentally on cellular phagocytosis. And macrophages, known for their robust phagocytic capacity, are a preferred cell type for this method. This approach fundamentally differs from using living cells as drug carriers. As AZCs lack cellular viability, they do not interact with encapsulated cargo. In contrast, living cells employed for drug loading may inevitably react with therapeutic agents, potentially leading to cargo inactivation or premature release^4^. Numerous studies have demonstrated the edge of the application of AZCs for intracellular cargo loading. For instance, a Trojan-like delivery approach was utilized by initially internalizing oncolytic viruses (OVs) into tumor cells *via* CD46-mediated endocytosis. Cryogenic shock treatment was then applied to neutralize the pathogenicity of tumor cells, resulting in the generation of “zombie” tumor cells loaded with OVs (LNT-Ad11). Studies have shown that LNT-Ad11 enhance OVs concentration by over 110 times compared to direct injection of natural OVs in lung metastasis progression^67^. After macrophages internalized magnetic iron (III) oxide nanoparticles, the resulting intracellular gelation, maintained the presence of CD163 (scavenger receptor) and CD206 (mannose receptor) on the cell membrane. These receptors are indispensable for microorganism recognition and binding. By applying an external magnetic field, microbial-bound cell particles can be isolated from complex biological environment, enabling convenient identification and collection of broad-spectrum microorganisms such as bacteria and fungi^68^. Intracellular loading strategies aim to preserve cell surface protein integrity and make use of inherent cell targeting capabilities to address issues related to inadequate drug accumulation. However, the effectiveness of these strategies is influenced by cell type and the properties of the delivery cargoes, the size and hydrophilicity of which could impact drug loading efficiency. Some cells with low phagocytic activity may be unable to achieve high drug loading efficiency 69, 70, 71. Thus, intracellular loading strategy is more suitable for cells with strong phagocytic ability or cargoes that can easily enter cells.

#### 3.2.2 Extracellular loading

Extracellular cargo loading onto AZCs is primarily achieved through method such as physical adsorption^72^, covalent coupling^73^, host-guest interaction^74^ or specific ligand-receptor interaction^75^. This approach enables targeted drug delivery resembling a “cell backpack” or “hitchhiking” mechanism^76^. Commonly loaded extracellular cargo includes nanodrugs, peptides, and probiotics. A key advantage of extracellular loading is the low incidence of undesired interactions between the carrier cell and the cargo. Physical adsorption typically relies on electrostatic attraction between the negatively charged surface of AZCs and positively charged cargo surfaces. This process avoids the tedious processes for complex chemical modifications and proceeds under mild conditions, thereby effectively preserving the conformational integrity and bioactivity of sensitive biomolecules. The charge-driven adsorption mechanism is not only operationally straightforward but also maintains the biological activity of the loaded therapeutics, making it a suitable strategy for loading macromolecules such as proteins and nucleic acids. For instance, cell vectors (referred to as LNT cells) that preserved the cell surface glycoprotein CD44 and cellular structure were developed after the liquid nitrogen cryopreservation shock treatment of KARS mutant non-small cell lung cancer (NSCLC) A549 cells. The LNT cells achieved lung-targeted drug delivery by passively lodging in lung capillaries and enhanced the targeting efficiency through CD44-mediated cell-to-cell interactions and adhesion. The liposome containing a cyclin-dependent kinase 4 (CDK4) gene knockout plasmid was embed onto LNT cells through electrostatic interactions. It was observed that the lung accumulation of the CDK4 gene knockout plasmid increased approximately fourfold compared to a traditional lipid nanoparticle formulation. The lipid nanoparticle formulation attached to zombie cells *via* physical adsorption leveraged both active and passive targeting mechanisms to effectively and precisely transport gene knockout plasmids to the lungs^66^.

Another approach involves attaching drug-loaded nanocarriers to the cell membrane through covalent bonding. Compared to physical adsorption, this covalent conjugation method offers superior stability, ensuring that therapeutic payloads remain securely anchored to APCs throughout systemic circulation until reaching target sites. Click chemistry was utilized to covalently attach sonosensitizer-loaded liposomes to genetically modified APC-4T1 cells. This method preserved the expression of costimulatory molecules (CD40, CD80/CD86) and major histocompatibility complex class I (MHC-I) on the surface of the resulting complex, termed DPNLs, even after subjecting the cells to cryogenic shock. In a murine model of triple negative breast cancer, DPNLs leveraged cell surface costimulatory molecules to trigger T (Treg) cell activation and proliferation in tumor draining lymph nodes (TDLNs), leading to tumor growth inhibition. Furthermore, in combination with liposome-encapsulated drugs, DPNLs demonstrated synergistic effects in achieving anti-PD-L1 activity and impeding tumor lung metastasis progression^77^.

*β*-Cyclodextrin (*β*-CD) is widely utilized for molecular recognition *via* host-guest interactions thanks to its exceptional biocompatibility and versatile guest binding properties 78, 79, 80. This host-guest interaction method offers a distinct strategy for engineering AZCs by employing the supramolecular recognition between *β*-CD and adamantane to reversibly anchor therapeutic cargo onto the cell surface without damaging membrane integrity. For instance, an artificial host-guest interaction-mediated bio-orthogonal supramolecular cell coupling approach was implemented. Initially, by leveraging the strong affinity between *β*-CD and adamantane/its derivatives, DSPE-PEG-CD was inserted into the membrane of living macrophages through lipid interactions. This led to the generation of *β*-CD-modified macrophages (CD-MA), which were subsequently subjected to cryogenic shock for 12 h to produce *β*-CD-modified “zombie” cells (CD-ZMA). These cells were then co-cultured with liposomes modified with adamantane. As a result, quercetin-loaded liposomes were effectively transported to inflammatory tissues in a “hitchhiking” way facilitated by the host-guest interaction between *β*-CD and adamantane^64^.

Receptors‒ligands interaction is the main basis for the biological effects of cell membranes, and it also provides an efficient and feasible approach for extracellular cargo loading on the surface of AZCs 81, 82, 83. This approach is characterized by its high specificity and excellent biocompatibility, as it capitalizes on inherent biomolecular recognition mechanisms to achieve precise cargo positioning while preserving the native conformation and functionality of membrane proteins. Specifically, this method was employed to convert macrophages into AZCs with hydrogels as their cytoskeletal support (GPM). By leveraging Toll-like receptors on the GPM membrane to selectively bind lipopolysaccharides (LPS) on probiotic membranes, probiotics were immobilized onto the GPM surface. This approach utilizes the inflammatory factor receptors on GPM surfaces to respond to elevated ligand concentrations in inflamed tissues, thereby promoting targeted accumulation at colitis sites. Moreover, the specific receptor-ligand interactions notably extend the probiotics’ residence time in the gut^84^. By maintaining the cell structure and membrane proteins, the inherent targeting capabilities of cells surpass those of targeting molecules. Subsequent engineering modifications endow synthetic “zombie” cells with efficient drug-carrying capacities^85^. These distinctive features enable AZCs to outperform traditional nanodrugs in various aspects.

### 3.3 Immunomodulation

#### 3.3.1 Innate immune regulation

AZCs, serving as a novel delivery vehicle combining inherent targeting capabilities with engineered features, have shown remarkable efficacy in targeted drug delivery. Moreover, they have been broadly applied in immunomodulation and neutralization of cytokines/toxins^86^^,^^87^. The immunomodulatory potential of AZCs stems from two core characteristics: their effective retention of source cell immune molecules (*e.g.*, co-stimulatory molecules, antigen-presenting complexes), coupled with a cell membrane that serves as an ideal platform for loading engineered immunomodulators. In contrast to conventional immunomodulators, AZCs achieve precise spatiotemporal control of immune activation and tolerance through synergistic physical-chemical approaches^88^. Additionally, AZCs offer significant benefits in terms of surface antigen concealment, pathogen eradication, regulation of the microenvironment adaptation and possess the ability to evade immune cell clearance while retaining natural targeting capabilities.

Although the immunosuppressive mechanisms within the tumor microenvironment (TME) remain unclear, there are several potential mechanisms recognized by researchers now. These include the recruitment of immunosuppressive cell populations, the secretion of immunosuppressive cytokines and metabolites, the upregulation of immune checkpoints, the downregulation of immunogenic antigens, and the inhibition of antigen presentation. These various mechanisms are intrinsically induced by the immunosuppressive microenvironment 89, 90, 91. In light of this, a novel therapeutic approach involving “zombie” macrophages was introduced to overcome the constraints imposed by the immunosuppressive TME on macrophage therapy and to activate innate immunity. Conventional macrophage adoptive transfer therapy is frequently restrained by the immunosuppressive TME, which leads to the conversion of the M1 phenotype to the M2 phenotype, consequently yielding no therapeutic effect^92^^,^^93^. However, by combining cryo-shocked M1 macrophages (CSMs) with the nano-drug CLNDN, the immunosuppressive TME barrier was effectively circumvented. CSMs maintain their M1 polarized phenotype and resist phenotypic reversal induced by the immunosuppressive microenvironment. CSMs drive tumor-associated macrophages (TAMs) towards a sustained M1 immunostimulatory state, enhance CD47 expression in tumor cells, and facilitate targeted CLNDN delivery. Additionally, CLNDN is employed to induce apoptosis specifically in tumor cells. This strategy effectively prevents secondary phenotype reversal caused by the microenvironment, preserving the therapeutic potential of cell therapy and overcoming constraints imposed by immunosuppressive microenvironment^94^.

#### 3.3.2 Antigen presentation and adaptive immune regulation

DCs, the strongest APCs *in vivo*, primarily facilitate adaptive immunity through their superior antigen presentation capacity 95, 96, 97. However, the antigen presentation capacity of DCs is influenced by the microenvironment. In immunosuppressive environments like tumor tissues, the expression of MHC-I and co-stimulatory molecules CD80/CD86 on the cell surface is downregulated, impeding the mediation of adaptive immunity^98^^,^^99^. Compared to conventional cell membrane-coated nanoparticles, artificial “zombie” DCs exhibit more comprehensively immunomodulatory functions. While membrane coating technology can transfer DC membranes onto synthetic nanoparticle surfaces, the extraction process inevitably leads to the loss or spatial conformational alteration of key signaling molecules, such as MHC‒peptide complexes. This inherent limitation restricts their ability to fully mimic natural APCs.

Through the application of cryogenic shock and intracellular hydrogelation, “zombie” DCs are artificially generated, preserving MHC-I, CD80/CD86, and damage-associated molecular patterns (DAMPs) on their membrane surface, and a versatile artificial antigen-presenting cell (aAPC) platform is therefore established. This technology rapidly and non-invasively converts cells into aAPC platform. The combined effects of (1) CD80-CD28 and MHC-I/TCR interactions and (2) target antigen peptide presentation collectively stimulate specific T-cell generation, modulate immune responses, and facilitate the development of adaptive immunity^100^. A tumor vaccine utilizing AZCs (LNT) loaded with an immune adjuvant was developed to achieve antigen-specific immunotherapy through co-delivery of antigen and adjuvant. This approach demonstrated prolonged *in vivo* retention, facilitating the development of cellular immune memory, enhancing T cell expansion, and improving anti-tumor efficacy^101^. Additionally, the aAPC platform exhibited favorable non-specificity and could be customized for antigen-specific immunity against particular antigens^102^.

In other studies, researchers have utilized AZCs to concurrently modulate innate and adaptive immunity in immune response processes. In cancer ultrasound immunotherapy, the limited efficacy is attributed to the absence of co-stimulatory molecules and MHC-I expression on tumor cells. To address these problems^103^^,^^104^, APC-like tumor AZCs developed through cryogenic shock treatment effectively maintained costimulatory molecules on tumor cells and enabled engineering capabilities. In this investigation, DNPLs were synthesized by crosslinking liposomes containing sonosensitizers after cryogenic shock of transfected APC-like tumor cells. It was found that these DNPLs could enhance MHC-I-independent natural killer cell (NK)-mediated innate immunity and T cell-mediated adaptive immunity^77^. In summary, AZCs can modulate adaptive immune responses through the preservation or reconfiguration of co-stimulatory signals and antigen-presenting molecules.

### 3.4 Cytokine/toxin clearance

The sequestration of membrane protein molecules of AZCs enables them to exhibit a distinctive capability in eliminating excess cytokines and biotoxins, a process dependent on specific binding facilitated by surface receptors on these cells^105^^,^^106^. Immune cells, such as neutrophils and macrophages, possess numerous Toll-like receptors (*e.g.* TLR2 and TLR4)^107^ and a raft of inflammatory factor receptors (*e.g.*, IL-1R and TNF-*α*R) on their cell membranes^108^. These receptors efficiently bind to corresponding cytokines in pathological conditions, leading to cytokines clearance. Biomimetic nanoparticles derived from neutrophil membranes have been investigated for their ability to target inflammatory sites and eliminate pro-inflammatory cytokines through cell membrane receptors of CXCL4, IL-1*β* and TNF-*α*, which showed promising therapeutic potential for conditions like rheumatoid arthritis (RA)^109^.

The process of extracting cell membranes involves multiple steps, including cell membrane disruption, membrane separation, and purification. This procedure is not only technically complex but also prone to cause the loss of functional membrane proteins. In contrast, the preparation of AZCs—using cryogenic shock as an example—centers on a single-step treatment of intact cells, eliminating the laborious independent membrane extraction and reconstruction steps. Despite the limitations associated with cell membrane applications, many researchers have leveraged membranes with preserved surface proteins as cytokine scavengers. For instance, neutrophil membrane-coated nanoparticles serving as scavengers for inflammatory cytokines (IL-1*β* and TNF-*α*), achieving a clearance rate of approximately 75%^107^. In a separate study, cryo-shocked AZCs were utilized as scavengers for cytokines/endotoxins (IL-1*β*, TNF-*α*, IL-6, and LPS), attaining a clearance efficiency of about 70%^27^. Thus, given their comparable efficacy in clearance, AZCs emerge as an optimal choice for cytokine/toxin elimination, combining a streamlined preparation process with robust functional performance^110^.

**Figure 2:**
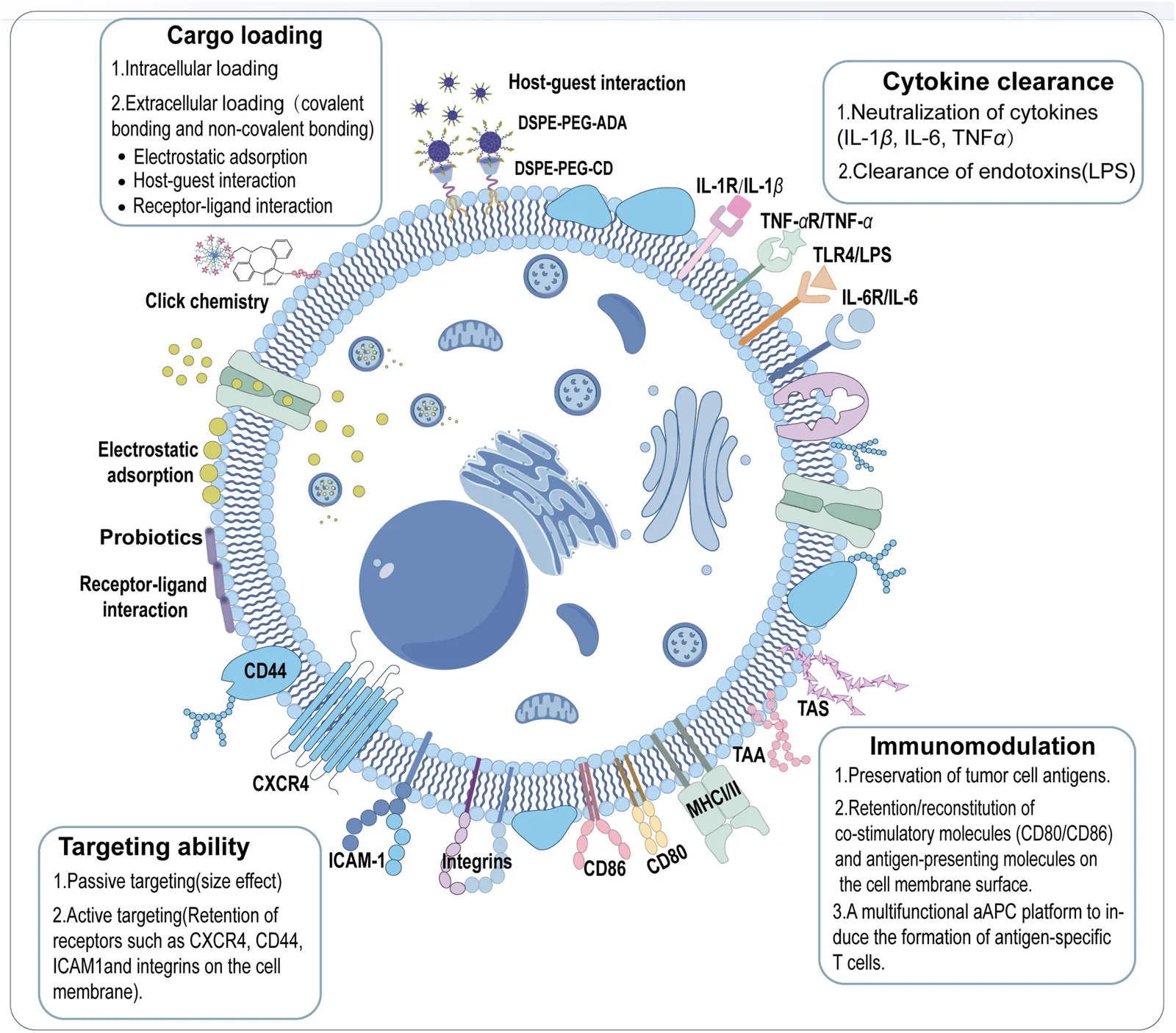
AZCs immobilized by various physicochemical methods exhibit unique biological properties and advantages for use in the treatment of disease, such as: drug delivery ability, tissue targeting capability, immunomodulatory potential, and cytokine/toxin scavenging ability.

Meanwhile, AZCs generated using intracellular hydrogelation also possess cytokine-clearing capabilities. Intracellular gelatinized macrophages (GMs) were engineered with this approach, which retained higher cell membrane receptor activity compared to conventional methods. GMs could eliminate excessive inflammatory factors in the inflammatory microenvironment through receptor–ligand interactions as well as selectively bind to inflammatory cells as immune cell carriers, effectively inhibiting the progression of arthritis^48^. Wang et al.^111^ demonstrated the neutralization of coxsackie virus B3 (CVB3) through receptor-ligand interactions, such as CAR and CD55, using endo-gelled cardiomyocytes (GCs). The intact cellular structure of GCs enabled them to serve as carriers for CVB3, effectively preventing CVB3-induced viral myocarditis (VMC). Due to their distinctive biological properties, targeted drug delivery capability, immune modulation potential, and capacity for cytokine/toxin clearance (Fig. 2), AZCs have been extensively applied in various medical conditions. In the next section, this review will systematically examine the therapeutic effect of AZCs in a range of diseases including tumors, inflammatory disorders, thromboembolic conditions, cardiovascular ailments, and bacterial infections.

## 4 AZCs for disease applications

**Figure 3:**
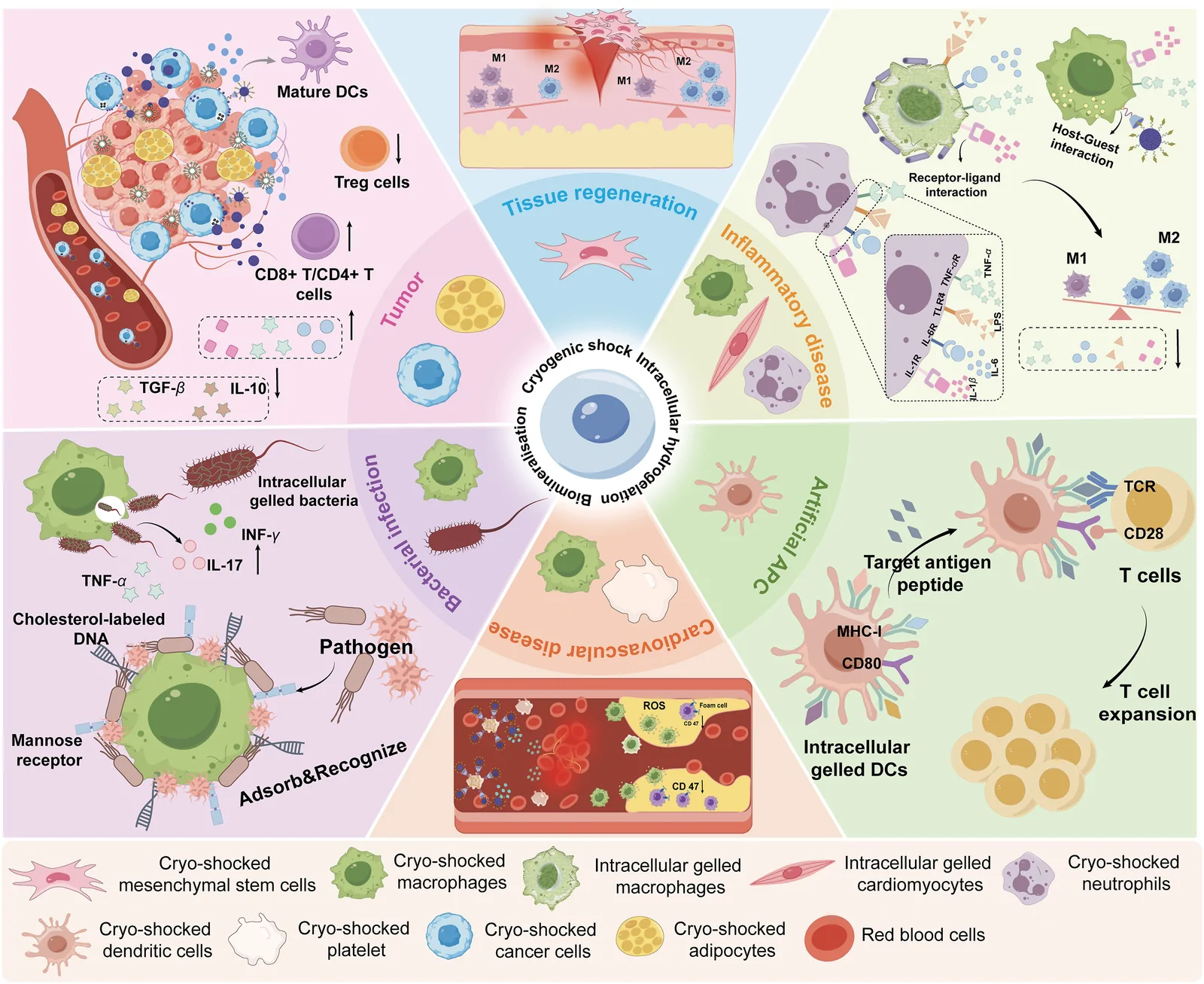
Application of AZCs in disease treatment. AZCs demonstrates its multifaceted clinical potential by enabling targeted therapeutic interventions in fields such as oncology, inflammatory diseases, and tissue regeneration.

AZCs represent a significant innovation in cell therapy and demonstrate broad therapeutic applicability across diverse diseases due to their unique biological properties and engineered features. These biomimetic vectors eliminate native cellular proliferation and viability while preserving intact membrane surface molecules, thereby enabling innovative solutions to overcome drug delivery barriers, modulate immune microenvironments, and achieve sustained tissue repair. These capabilities originate from three core attributes: precise targeting conferred by their bionic interface, high drug-loading capacity facilitated by cross-linking networks, and prolonged *in vivo* circulation resulting from metabolic termination. Current applications span tumors, inflammatory disorders, thromboembolic conditions, cardiovascular diseases, and bacterial infections, indicating therapeutic potential exceeding that of traditional nanomedicines. Recent disease treatment applications of AZCs were summarized based on cell type classification (Table 2). The mechanisms by which AZCs are used to treat various diseases were also displayed (Fig. 3).

### 4.1 Tumors

#### 4.1.1 Tumor vaccine

Tumor vaccine is a therapeutic approach that leverages the immune system to combat cancer cells by stimulating the body's adaptive immune response through the delivery of tumor-associated or tumor-specific antigens, aiming for the precise eradication of tumor cells. These vaccines are categorized into therapeutic vaccines and preventive vaccines based on their intended targets 112, 113, 114. In general, the effectiveness of tumor vaccine could be affected by poorly immunogenic and rapid clearance of tumor antigens and adjuvants. Within the TME, artificial “zombie” cancer cells, serving as integrated reservoirs of diverse antigens, can be employed to trigger broad immune responses based on complete antigen presentation^115^. For instance, AML is a hematologic malignancy characterized by a grim prognosis^116^. Ci et al.^16^ discovered that AML cells possess a natural homing ability to the bone marrow based on adhesion receptors CD44 and CXCR4. Exploiting this trait to address the limitation of conventional chemotherapy drugs in targeting the bone marrow, they utilized cryogenic shock technology to generate “zombie” AML cells capable of encapsulating therapeutic agents. In murine models of AML, DOX-loaded LNT cells administration followed by adjuvant demonstrated superior anti-tumor efficacy as therapeutic vaccine by stimulating anti-tumor immune reactions, extending the survival rate of AML-afflicted mice up to 50% at Day 60. Meanwhile, the unset of AML in mice was forcefully controlled by preimmunizing with LNT cells and adjuvant, and 71% of C1498 cells-challenged mice were tumor free at Day 90^117^. Furthermore, LNT cells, as drug carriers, can simplify the separated administration procedures and prevents fast clearance of vaccenic components by loading cancer adjuvants. For example, Chen et al.^101^ introduced a tumor vaccine using liquid nitrogen cryopreserved B16F10 cells (LNT cells) as carriers containing adjuvant R837. This approach facilitated the simultaneous co-uptake of antigen and adjuvant and thereby extended the *in vivo* retention time of the vaccine. The combined action of both components resulted in enhanced *in vivo* prophylactic efficiency and improved survival rate to 50% on Day 60 in B16F10 cell-challenged mice.

**Figure 4:**
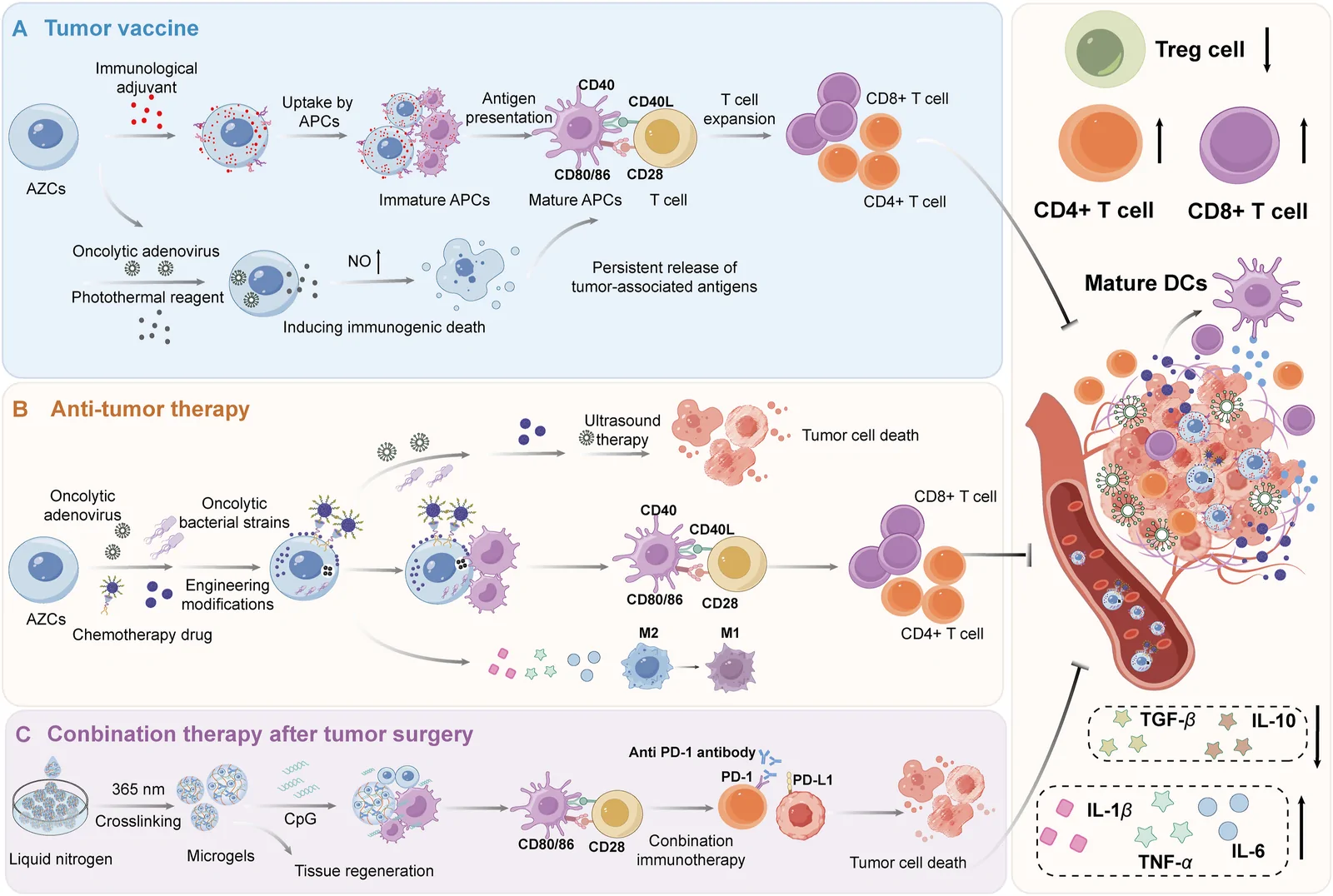
This diagram primarily summarizes the mechanisms of AZCs in tumor applications, which are divided into three main sections. (A) In the first section, AZCs are utilized as cancer vaccines. These vaccines typically consist of AZCs combined with immune adjuvants, where AZCs function to provide TAAs. Together, they activate anti-tumor immune responses to achieve therapeutic effects against tumors. (B) In the second section focusing on anti-tumor therapy applications, AZCs derived from tumor cells, adipocytes, macrophages, and neutrophils, following liquid nitrogen cryogenic shock and engineered modifications, are employed for anti-tumor treatment. This approach leverages the unique characteristics of each cell type, such as the size effect of cells, the active recruitment of adipocytes to the TME for targeted delivery, and the inflammatory factor-neutralizing capabilities of macrophages and neutrophils. These mechanisms collectively contribute to activating anti-tumor immune responses and eliminating tumor cells. (C) The third section describes the use of a microgel encapsulating AZCs, immune adjuvants, and immune checkpoint inhibitors for combination therapy following tumor surgery. This strategy aims to achieve synergistic effects by activating immune responses and promoting tissue repair.

Although the vaccine strategy based on LNT cells has achieved certain results, researchers are still constantly exploring more efficient and stable methods for preparing whole tumor antigen vaccines^118^. In addition to the aforementioned methods, two strategies have been developed for the efficient preparation of whole tumor antigen vaccines by integrating physical and chemical techniques: cryo-silicification technology and the FeCl_3_ chemical induction method. The fundamental principle of these approaches involves the rapid immobilization of cancer cells *in situ* through a biomineralization process, transforming them into whole tumor vaccine^119^. In the realm of tumor vaccine, innovative approaches have been successfully implemented. For instance, Qi et al.^58^ developed a thermostable vaccine that maintains tumor antigen integrity by combining cryopreserved tumor cells with engineered pathogen-associated molecular patterns (PAMPs). This strategy induced robust antigen-specific T cell immunity, leading to complete tumor eradication in ovarian cancer models. Similarly, Zhang et al.^54^ introduced a novel magnetic sculpture-like tumor cell vaccine (MASK) that induced rapid biomineralization, conferring magnetism to tumor cells *via* high FeCl_3_ concentrations. Through magnetic field-guided localization to tumor sites, MASK effectively triggered *in situ* anti-tumor immune responses, resulting in significant tumor growth inhibition. Furthermore, combining MASK with PD-1 inhibitors further enhanced therapeutic efficacy (Fig. 4A). All above highlighted the potential values of AZCs as both a therapeutic and preventive tumor vaccine, and the orderly combination of AZCs with therapeutic drugs and adjuvants reflected advanced cell-based tumor vaccine strategy.

In addition to using AZCs as antigen sources, they can also serve as carriers to deliver immunogenic death-inducing agent and locally produce tumor-associated antigens (TAAs) *in vivo*. A novel approach was proposed for the efficient preparation and sustainable delivery of TAAs. This method involved subjecting human glioblastoma cells U251 infected with oncolytic adenoviruses (OAs) to cryogenic shock treatment so as to establish an oncolytic adenovirus library (OAR). Cryogenic shock treatment enabled the cancer cells loaded with OAs to constantly release TAAs and OAs within the TME, leading to tumor lysis. Consequently, the released tumor antigens activated dendritic cells, promoted infiltration of CD8^+^ T cells, increased cytokine secretion, reduced regulatory T cell infiltration, and ultimately elicited a robust anti-tumor immune response^120^. Recurrence and metastasis pose significant challenges in current tumor clinical treatment^121^^,^^122^. Another novel approach involving the supramolecular assembly of “ice cells and fire nanomedicines” was devised to bolster the anti-tumor immune response and impede the progression of distant tumors and lung metastasis with a single treatment. This method entailed utilizing cancer cells shock-frozen with liquid nitrogen (LNF) as a source of TAAs. These LNFs were coupled with nanoparticles loaded with a nitric oxide (NO) precursor that exhibited a photothermal effect through host-guest interactions. Upon photothermal induction, this supramolecular cell coupling platform could generate high concentrations of NO at the tumor site, enhancing drug penetration in tumor tissues and markedly reducing the recurrence rate of *in situ* tumors^123^. These innovative cryo-shock approaches thus transform treated tumor cells into an *in situ*, sustained-release vaccine, effectively overcoming key hurdles of immunogenicity and antigen supply in cancer treatment (Fig. 4B).

#### 4.1.2 Anti-tumor therapy

Malignant melanoma is acknowledged as the most aggressive type of skin cancer, characterized by high invasiveness and notable metastatic potential^124^^,^^125^. Though lung metastasis stands out as a prevalent form of metastasis in advanced melanoma, current therapeutic options remain limited in efficacy and burdened by high costs. To address these challenges and hinder lung metastasis, Li et al.^126^^,^^127^ devised a biomimetic system, using harnessed adipocytes^128^, pivotal in tumor cell invasion and metastasis, as carriers for drug delivery. These adipocytes were rendered inactive through cryopreservation with liquid nitrogen (CsA) while their protein-mediated biological functions were preserved. *In vivo*, tumor cells actively attracted CsA, which, due to its size, became entrapped in the lungs, thereby enhancing drug accumulation in this organ. CsA taken by tumors could disrupt the interaction between tumor cells and normal adipocytes, and co-deliver triptolide palmitate (pTP) the photosensitizer Ce6 to combine photodynamic therapy (PDT) and pTP activates the caspase, promoting apoptosis in tumor cells, consequently impeding tumor progression and achieving metastasis inhibition rates of 88.83 ± 1.80%^129^. This biomimetic strategy, therefore, leverages the tumor's own biological mechanisms to achieve targeted delivery and represents a significant advancement in the pursuit of more effective and precise therapies against metastatic melanoma.

AZCs have also been utilized in the treatment of lung cancer. Zang et al.^130^ developed immunogenic AZCs with ultraviolet irradiation and cryogenic shock on a mouse lung cancer cell line (LLC), creating UV-cryo cells. These UV-cryo cells were loaded with anti-tumor drugs, 10-hydroxycamptothecin (HCPT) and Quillaja saponin-21 (QS-21), and due to their size, they were passively targeted to the lungs where they were trapped by pulmonary capillaries. This passive process enhanced antigen uptake and presentation by antigen-presenting cells, eliciting an effective immune response as a cancer vaccine. The combination of chemotherapy and immunotherapy resulted in synergistic effects. Furthermore, thanks to their active targeting capabilities, AZCs are commonly employed for drug delivery to specific tumor sites. Liu et al.^66^ developed LNT cells for delivering CRISPR-Cas9 liposomal nanoparticles with cryogenic shock. This delivery system promoted lung targeting through passive entrapment in pulmonary capillaries and interactions with endothelial cells. In a mouse model of NSCLC, lipid nanocarrier technology (LNT) demonstrates increased lung accumulation compared to lipid nanoparticles alone. Moreover, the expression of CDK4 is significantly reduced in tumors. Notably, CRISPR-Cas9 editing-induced CDK4 ablation leads to the demise of KRAS-mutant NSCLC cells, thereby prolonging the survival of NSCLC mice. These studies collectively highlight the versatility of AZCs as a powerful platform for enhancing both targeted drug delivery and innovative immunotherapeutic strategies against lung cancer (Fig. 4B).

In recent years, intracellular hydrogel technology has been utilized to create dead and internally gelatinized bacteria. Le et al.^131^ referred to these endo-gelatinized bacteria as cyborg cells, and their integrity of genetic material, flow, functional cell membrane interfaces, and active metabolic pathways were maintained. Their study showed that cyborg cells possessed the capability to infiltrate cancer cells *in vitro*. Additionally, Khan et al.^132^ found that macrophages could internalize cyborg cells based on previous research, which made it possible to utilize these novel cyborg pathogens for various biomedical applications, including drug delivery and immunotherapy. Collectively, these advancements underscore a paradigm shift in biomedicine, where engineered cellular “cyborgs”—whether derived from host immune cells or pathogens themselves—are being repurposed as sophisticated, multifunctional platforms that blur the line between biological entity and diagnostic/therapeutic tool, opening new frontiers in the fight against infectious diseases and cancer.

#### 4.1.3 Combination therapy after tumor surgery

Preventing tumor recurrence and metastasis as well as promoting tissue regeneration are essential aspects of postoperative treatment for malignant tumors^133^. Following tumor resection, combined chemotherapy and radiotherapy constitute a standard preventive strategy against recurrence, yet this approach inherently carries significant side effects and demonstrates limited therapeutic efficacy^134^. Immunotherapy has emerged as a promising post-oncology treatment strategy^135^. Nevertheless, the repeated systemic administration of immunotherapeutic agents can lead to uncontrolled immune rejection and adverse reactions^136^. To address these challenges, Kuang et al.^137^ have introduced a novel microgel co-loaded with cryogenic shock-treated cancer cells, immune adjuvants and immune checkpoint inhibitors. It has been proved that this innovative microgel has the ability to recruit dendritic cells *in vivo* and activate them locally, thereby eliciting a potent anti-tumor immune response. Research has shown that microgel effectively inhibits tumor recurrence and metastasis in an *in-situ* breast cancer mouse model due to its favorable biocompatibility and appropriate degradation properties, facilitating normal cell adhesion and growth to support tissue reconstruction. AZCs have proven to be reliable in presenting TAAs across various anti-tumor treatments. Following cryogenic shock, these cells lose the viability and pathogenicity of the original tumor cells, becoming safer and offering more robust antigenic stimulation compared to inactivated tumor cells alone. Therefore, this strategy successfully transforms postoperative challenges into an opportunity for targeted healing and lasting immunity, highlighting the dual-function potential of AZC-based biomaterials in cancer therapy (Fig. 4C).

In summary, AZCs represent distinctive therapeutic promise in cancer vaccines, anti-tumor therapy, and post-tumor surgery recovery. Their unique value lies in their ability to serve as versatile antigen-presenting platforms that can be engineered for targeted drug delivery, potent and sustained immune activation, and even supporting tissue regeneration, thereby addressing multiple challenges in oncology.

### 4.2 Septicemia

**Figure 5:**
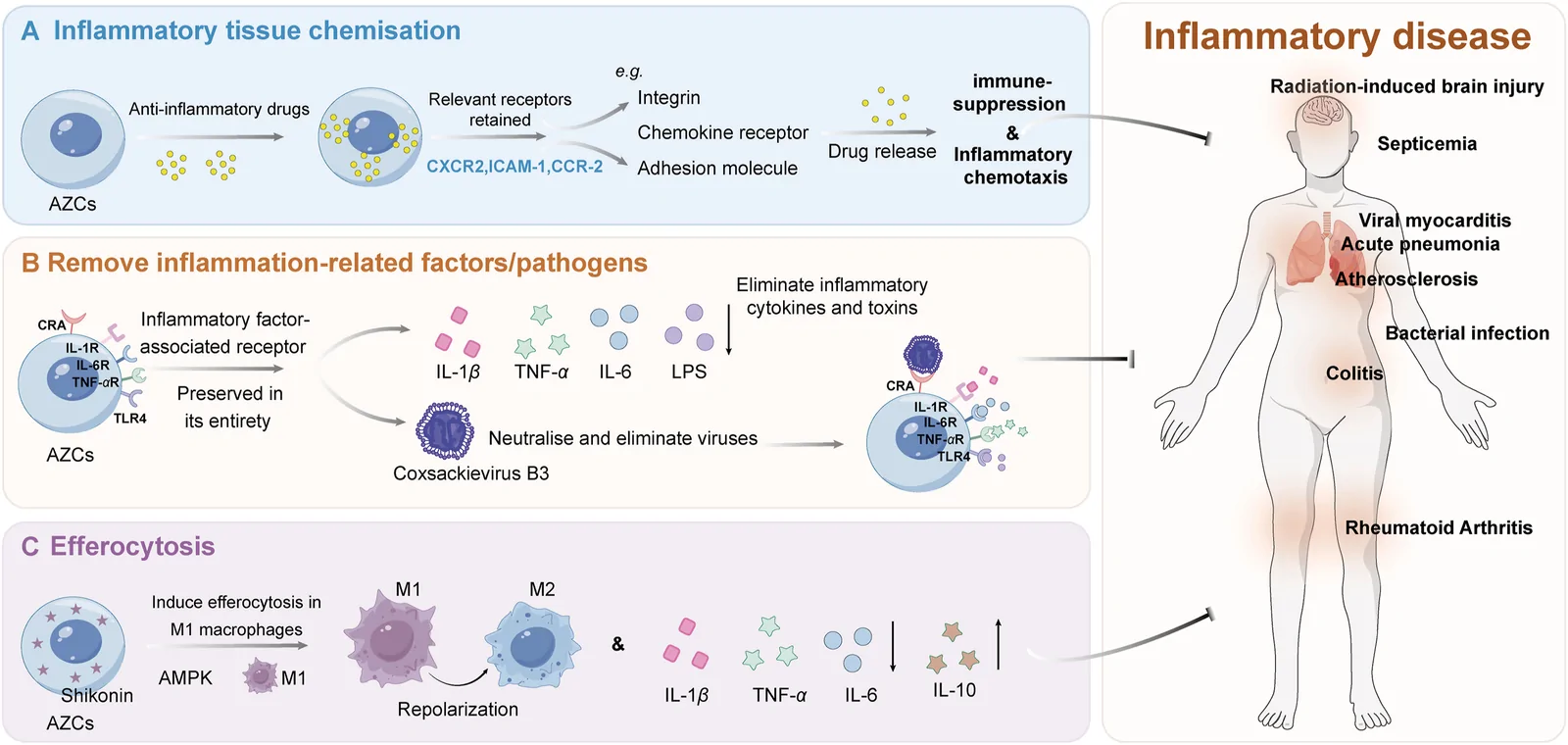
Mechanisms of AZCs in treating inflammatory diseases. (A) Inflammatory tissue targeting: AZCs utilizes specific receptors preserved on its cell membrane to mediate chemotaxis toward inflammatory sites. This enables the targeted delivery of anti-inflammatory agents directly to the loci of inflammation. (B) Clearance of inflammatory factors: AZCs employs remaining receptors for inflammatory factors on its surface to bind and effectively clear these mediators from the body. (C) Macrophage phenotype switching *via* efferocytosis: AZCs triggers efferocytosis in M1 macrophages, promoting the transition to the M2 phenotype. This shift from a pro-inflammatory to an anti-inflammatory state achieves suppression of inflammation.

AZCs have emerged as an innovative therapeutic platform for treating a spectrum of inflammatory diseases, including sepsis, acute pneumonia, RA, colitis, viral myocarditis, atherosclerosis, bacterial infections, and radiation-induced brain injury. Their therapeutic efficacy stems from three mechanistic pathways: (1) inflammatory tissue tropism, enabling targeted accumulation at disease sites (Fig. 5A); (2) clearance of pathogenic inflammatory mediators (Fig. 5B); and (3) induction of efferocytosis to resolve inflammation and promote tissue repair (Fig. 5C). By integrating these multifaceted actions, AZCs offer a potent strategy to restore immune homeostasis, addressing the core limitations of conventional anti-inflammatory regimens.

The fundamental pathology of inflammatory diseases is rooted in the excessive release of inflammatory mediators and tissue damage resulting from an imbalance in the immune response, potentially leading to a severe condition known as a “cytokine storm”. Conventional anti-inflammatory medications often lack precise targeting and may induce systemic immunosuppression, thereby posing risks of significant side effects^138^^,^^139^. A novel approach in the treatment of inflammatory diseases involves the development of an AZC bionic drug delivery vector. This vector is created using cryogenic shock and intracellular hydrogelation, aiming to preserve intact receptors and immune evasion properties on the cell membrane surface^140^. For instance, sepsis is a condition characterized by a systemic inflammatory response and the overactivation of cytokine storms. Research indicates that the systemic dissemination of LPS from the initial site of infection directly drives this severe immune dysregulation^141^. Gao et al.^27^ devised a comprehensive anti-inflammatory and endotoxin neutralization approach grounded in sepsis pathogenesis. By subjecting neutrophil membranes to cryogenic shock, they maintained the protein integrity and functions of CS-Neus membrane. This method demonstrated promising therapeutic efficacy in both LPS-induced sepsis mouse models and blood samples from clinical sepsis patients. CS-Neus exhibited the ability to efficiently eliminate inflammatory cytokines and LPS, attenuate the cytokine storm, reduce inflammation, and enhance the survival rate of septic mice. This innovative biomimetic strategy, which leverages the innate pathogen-sequestering function of immune cells, presents a highly targeted and potent therapeutic avenue for mitigating uncontrolled inflammation without inducing systemic immunosuppression.

### 4.3 Acute pneumonia

Building on the concept of using biomimetic systems for targeted anti-inflammatory therapy, this approach has been specifically adapted to address the challenges of treating acute pneumonia. Acute pneumonia, a respiratory infection caused by various pathogens, ranks among the leading global causes of mortality^142^^,^^143^. Many anti-inflammatory medications tested in clinical settings exhibit poor precision in targeting specific sites. Hence, enhancing the localization of anti-inflammatory agents at sites of inflammation is vital for the effective treatment of acute pneumonia^144^^,^^145^. Xu et al.^64^ devised a supramolecular macrophage-nanopharmaceutical conjugate for delivering drugs to the lungs to address acute pneumonia in mice. This involved linking adamantane-modified liposomes to *β*-CD-modified artificial macrophages through host-guest interactions. The supramolecular conjugate served as a stable vehicle for drug delivery, enabling the transportation of drugs to inflamed tissues. This supramolecular strategy exemplifies a significant advancement in targeted therapy, as it synergistically combines the innate inflammatory-tropism of macrophage membranes with the high drug-loading capacity of nanocarriers, offering a promising path to improve drug accumulation at the infection site while minimizing systemic side effects.

### 4.4 Rheumatoid arthritis (RA)

The application of biomimetic zombie cell technology further extends to chronic autoimmune conditions, demonstrating its versatility in modulating complex inflammatory networks. RA is a common autoimmune disease characterized by synovial inflammation in joints^146^^,^^147^. At present, clinical treatment is mostly aimed at controlling inflammation, and antirheumatic as well as non-steroidal anti-inflammatory drugs are mostly used to block inflammation^148^. As pathological inflammation is synergistically controlled by a complex network of cytokines and chemokines, simple inhibition of inflammation alone has little effect on disease treatment^149^^,^^150^. Li et al.^60^ demonstrated that targeted efferocytosis reprograms pro-inflammatory M1 macrophages into an anti-inflammatory M2 phenotype. Subsequently, these M2 macrophages released anti-inflammatory mediators to re-establish immune homeostasis. In their study, they loaded the small-molecule anti-inflammatory drug shikonin (SHK) into liquid nitrogen cryo-shock-induced apoptotic macrophages (designated as DM/SHK). DM/SHK continuously converted M1 macrophages in infiltrating synovium to M2-type to restore immune balance, inhibit proliferation and infiltration of synovium, prevent cartilage and bone erosion, and restore immune homeostasis of RA mice with inflammation.

AZCs, created using intracellular hydrogelation where the hydrogel forms a three-dimensional network mimicking a cytoskeleton, serve as effective scavengers of inflammatory cytokines. Gao et al.^48^ engineered intracellular GMs that retained their cell membrane structure. These GMs efficiently neutralized a variety of inflammatory cytokines, mitigated joint damage and inhibited arthritis progression in a collagen-induced rat arthritis model, exerting potent anti-inflammatory effects. Consequently, GMs hold significant promise for modulating inflammation and immune homeostasis. In summary, these studies underscore the dual therapeutic power of AZCs in autoimmunity: they can not only actively reprogram the local immune microenvironment, as seen in RA, but also function as passive, high-capacity decoys to systemically quench inflammatory signals, offering a multifaceted platform for restoring immune balance.

### 4.5 Colitis

The therapeutic potential of AZCs platforms is also being demonstrated in the complex milieu of gastrointestinal inflammation, another type of refractory autoimmune disease, offering a novel strategy to address the multi-factorial pathology of inflammatory bowel disease (IBD). IBD is a persistent gastrointestinal inflammation characterized by the involvement of multiple inflammatory cytokines that perpetuate the damage to intestinal epithelial cells. Various neutralization strategies targeting different cytokines have been proposed to address this perplexing pathogenesis^151^^,^^152^. Wang et al.^153^^,^^154^ have introduced a novel approach using endo-gelled macrophages (GPMs) as targeted carriers for probiotics. These GPMs, which preserved macrophage membrane surface receptors, such as scavenger receptors, TLRs and cytokine/chemokine receptors, prolonged probiotic intestinal retention to restore gut microbiota homeostasis. The conjugation of GPMs with probiotics has demonstrated efficacy in mitigating inflammatory cytokines, modulating oxidative stress, inflammation levels, promoting gut barrier repair, balancing gut flora, and regulating acid metabolism. These interventions collectively contribute to reinstating intestinal stability in colitis-afflicted mice^84^. This GPM-probiotic synergy represents a paradigm shift in IBD treatment, moving beyond simple cytokine blockade to actively restoring gut ecological balance through targeted, multi-mechanistic intervention.

### 4.6 Viral myocarditis

The utility of AZCs-based inflammatory factor neutralization strategy has already extended beyond systemic inflammation and autoimmunity to the precise neutralization of viral pathogens in critical organs. VMC is primarily caused by CVB3 infection. Current treatment for VMC typically involves dependence on the use of broad-spectrum antiviral drugs. However, these drugs often have non-specific and off-target effects, resulting in severe side effects^155^^,^^156^. Wang et al.^111^ have introduced a novel approach to address this issue by developing a cellular sponge known as GCs through an intracellular hydrogel technique. GCs have shown the ability to specifically recognize and adsorb viruses *via* membrane receptors, thereby rendering the viruses incapable of infecting normal host cells. In a murine model of VMC, GCs attained remarkable immune evasion capabilities and effectively impeded the progression of CVB3-induced VMC. This innovative strategy offers a promising treatment approach for viral infections, not only targeting known viruses but also potentially addressing infections caused by mutated or unidentified viruses.

As a novel engineering approach, these “zombie” cells are designed to specifically target inflammatory sites, eliminate inflammatory cytokines and toxins to restore microenvironmental homeostasis. The functional stability and safety of AZCs are achieved through freezing and crosslinking techniques. In contrast to living cells, AZCs lack metabolic activity and do not elicit uncontrolled immune responses *in vivo*. Their preparation and storage requirements are simpler and cheaper than those of living cells, potentially facilitating their clinical translation for the treatment of diseases such as septic shock and RA.

### 4.7 Atherosclerosis

Building upon their success in oncology and immunology, the application of AZC technology is demonstrating significant promise in managing complex cardiovascular pathologies, particularly atherosclerosis 157, 158, 159. Atherosclerosis, a prevalent cardiovascular ailment, stands as a dominant global cause of mortality^160^. The progression of atherosclerosis is driven by the infiltration of immune cells into plaques, which leads to macrophages potentially transforming into detrimental foam cells, thereby inducing macrophage immune dysfunction^161^. In a recent study, a “Trojan Horse” bionic drug delivery system known as ZARMs was devised. This system was based on artificial “zombie” macrophages (Z-Møs) preserved in liquid nitrogen to regulate macrophage immune responses within atherosclerotic plaques. Reactive oxygen species (ROS)-responsive nanoparticles (AR-NPs) containing atorvastatin were incorporated into Z-Møs to create biomimetic drug delivery systems known as ZARMs. Following intravenous injection, ZARMs exhibited targeted migration towards atherosclerotic plaques. The elevated levels of ROS within the plaques triggered the breakdown of AR-NPs and leaded to the release of the encapsulated drugs. In a study involving atherosclerotic mice, ZARMs demonstrated efficacy in boosting macrophage immunity and suppressing CD47 expression on foam cells, thereby reducing inflammation and impeding the advancement of plaque formation^162^. The ZARMs system exemplifies a sophisticated next-generation therapeutic strategy, successfully merging the innate homing ability of macrophages with smart, stimulus-responsive drug release to actively modulate the local plaque microenvironment, thereby addressing the root causes of atherosclerosis progression.

### 4.8 Bacterial infection

Beyond therapeutic applications, the versatility of AZCs-based platforms extends to the critical area of diagnostics, offering innovative solutions for the rapid and precise detection of pathogenic threats^163^. Gui et al.^68^ designed an innovative pathogen detection system based on AZCs created through intracellular hydrogel technology. This system can recognize, capture, enrich, and detect various bacteria along with their secreted exotoxins. By leveraging the phagocytic capability of macrophages to internalize magnetic nanoparticles, the researchers engineered gel-encapsulated macrophages (GMøs) through light-induced crosslinking. Subsequently, they functionalized DNA sensing components onto the GMøs surface to fabricate DNA-GMøs intelligent pathogen detectors. These detectors not only efficiently captured and identified diverse pathogens but also enabled pathogen retrieval through external magnetic manipulation. In conclusion, this intelligent pathogen detector exhibited broad utility in bacterial analysis and holds promise for application in the diagnosis and management of infectious diseases.

### 4.9 Radiation-induced brain injury

The application of AZC technology further demonstrates its unique advantages in addressing the challenges of acute radiation-induced neurological damage, showcasing its potential for targeted therapy in highly sensitive tissues. Acute radiation syndrome (ARS), triggered by short-term, high dose ionizing radiation exposure, is a systemic disorder primarily affecting the central nervous system (highly sensitive), endocrine, circulatory, and reproductive systems. It induces oxidative stress and neuroinflammation, disrupts cerebral microvasculature and the blood‒brain barrier (BBB), ultimately causing neurological dysfunction^164^^,^^165^. Radiation-induced brain injury (RIBI) typically manifests as cognitive decline, with severe cases progressing to cerebral necrosis, atrophy, even secondary tumors. Conventional RIBI treatments exhibit limited efficacy and may increase hemorrhage risk^166^^,^^167^. Built on this foundation, cryo-shocked neutrophils (CS-Neu) were proposed as nanodrug delivery vehicles. This approach avoided inflammatory exacerbation and nanodrug decomposition associated with activated neutrophils. Furthermore, it leveraged receptors on the CS-Neu membrane surface for targeted delivery to inflammatory sites and brain enrichment, successfully transporting nanodrugs to exert therapeutic effects in the brain. Consequently, this method mitigates radiation-induced oxidative damage, neuroinflammation, and tissue damage, while simultaneously promoting BBB restoration and alleviating cognitive impairment in RIBI mice^168^. This CS-Neu-based strategy represents a breakthrough in neuroprotection, as it ingeniously transforms a key inflammatory cell into a safe and effective Trojan horse, enabling precise drug delivery across the compromised BBB to combat the multifaceted pathology of radiation-induced brain injury at its source.

### 4.10 Thromboembolic disease

Beyond their established roles in oncology and inflammatory diseases, AZCs are also being explored for thromboembolic diseases and tissue regeneration. Although antithrombotic therapy (including antiplatelet agents and anticoagulants) remains the primary treatment for vascular embolism, its therapeutic efficacy is often limited by poor targeting efficacy and adverse effects stemming from high dosages^169^. The targeted delivery capabilities of AZCs were also exemplified in the treatment of thromboembolic diseases, offering a solution to the limitations of conventional antithrombotic therapy. In a recent study, Quan et al.^170^ introduced a biomimetic drug delivery system that leveraged cryopreserved platelet-like particles (CsPLTs) for enhanced efficacy in targeting thrombi. CsPLTs maintained the structural integrity and surface protein composition of platelets, thereby facilitating precise thrombus localization. This innovative system involved the encapsulation of aspirin and thrombolytic agents within ROS-responsive liposomes modified with adamantane. Meanwhile, the attachment of these liposomes to *β*-CD-modified CsPLTs (referred to as CPSAT) was facilitated through host-guest interactions. Notably, CPSAT has demonstrated obvious thrombus-targeting capabilities and superior therapeutic outcomes in murine models of thromboembolic conditions, which represents a significant advancement in antithrombotic strategy, as it synergistically combines the innate homing ability of platelet membranes with intelligent, site-specific drug release, thereby maximizing therapeutic efficacy at the thrombus site while minimizing systemic side effects.

### 4.11 Tissue regeneration

The skin serves as a protective barrier for the subcutaneous tissue and plays a crucial role in maintaining the body's physiological equilibrium. However, it is susceptible to external damage and infections^171^. While minor skin injuries could heal within 7‒14 days, severe damage often leads to the formation of non-functional scar tissues^172^. That's why prompt healing of skin lesions becomes particularly important when they exceed a critical size^173^. Yang et al.^174^ employed cryogenic shock to generate umbilical cord mesenchymal stem cells (LNT-MSCs) that, despite undergoing cell death, retained their functions in expediting skin wound recovery. Research indicates that LNT-MSCs possess the ability to accelerate the healing of skin wounds, offering a novel approach to tissue regeneration. This approach represents a significant innovation in regenerative medicine, as it leverages the therapeutic cargo and signaling functions of stem cells in a stable, “off-the-shelf” AZC format, effectively bypassing the viability and safety concerns associated with living cell therapies while actively promoting functional tissue restoration.

In summary, AZCs, engineered through cryogenic shock, intracellular hydrogelation and biomineralization represent a transformative biomimetic platform with profound implications across bio-medicine. The overarching power of AZC technology lies in its ability to synergize the innate, evolved targeting mechanisms of biological systems with the controlled efficacy and safety of engineered materials, thereby offering a versatile strategy to overcome the limitations of poor targeting, systemic side effects, and complex manufacturing associated with conventional therapies. Ultimately, AZCs bridge a critical gap between synthetic nanomedicine and complex pathophysiology, heralding a new era of sophisticated, biomimetic interventions for a wide range of human diseases.

## 5 Outlook

Functionalized AZCs, created by cryogenic shock and intracellular hydrogelation and other techniques, offer innovative approaches for disease treatment and demonstrate unique therapeutic potential in different medical conditions. The original cell structure and membrane protein molecules endow these cells with bionic interface properties. Meanwhile, the development of stable storage methods for these cells has been achieved, alongside a comprehensive investigation of their distinctive biological attributes. Notably, AZCs have been observed to leverage cytokine receptors present on their membrane surfaces, enabling natural targeting by cytokines within tumor or inflammatory microenvironments. Moreover, the intact cellular structure of AZCs enables precise drug delivery to targeted sites. Beyond their drug delivery capabilities, these cells also show immune modulation and cytokine clearance functions, establishing a solid groundwork for future disease treatment applications.

In the field of disease treatment, AZCs have demonstrated remarkable efficacy in addressing various conditions such as tumors, acute pneumonia, RA, and atherosclerosis. With distinctive biological properties, these artificial cells function as highly efficient vehicles for targeting drug delivery, as well as agents for neutralizing cytokines and toxins. They represent a rare class of carrier materials combining both targeting and therapeutic functionalities. In cancer therapy, conventional chemotherapy drugs often elicit severe adverse reactions in healthy tissues when attacking tumor cells. Due to their affinity for the TME, AZCs can precisely transport drugs to tumor sites. Targeted drug delivery approaches utilizing AZCs not only enhance drug accumulation in tumor tissues and increase tumor cell lethality but also significantly reduce drug dispersion in normal tissues, thereby mitigating systemic toxicities^23^. AZCs, as bionic vectors, also offer distinct advantages in post-tumor surgery scenarios by effectively preventing recurrence and metastasis, which is attributed to their excellent biocompatibility. These cells are further enhanced by the incorporation of various therapeutic agents through surface or internal modifications, facilitating a synergistic treatment approach that elevates the efficacy of tumor therapy. By precisely targeting lesions, rebalancing immune responses, suppressing inflammatory mediators, and fostering tissue regeneration, these cells introduce novel therapeutic avenues for a range of diseases, underscoring their significant clinical prospect. Furthermore, AZCs should be categorized as biological agents, making their utilization in clinical studies further complicated. While the procedural requirements may be more intricate compared to trials involving single-component drugs, they are relatively less burdensome in terms of ethical review and supervision in contrast with studies involving living cells. For example, in China, carrying out clinical research on living stem cells has been plagued by an excessively long approval process and a low permission rate, which is not conducive to advancing stem cell clinical research, nor does it adequately consider the complexity, variability, and diversity of early clinical studies, making it even more challenging than submitting a new drug application. So, research groups from China have already adopted dead mesenchymal stem cells for IIT clinical research (led by researchers from medical institutions rather than by regulatory agencies; ongoing clinical trials: ChiCTR2300075243, ChiCTR2300075235, NCT06021067) to avoid the cumbersome application and review procedures of living stem cells research. The streamlined approval process could reduce time and cut costs during the research application phase, making it faster to move from bench to besides.

Despite the unique advantages of AZCs in disease treatment, various technical and biological challenges in clinical application have also occurred. One major concern is the long-term safety. Once introduced into the human body, AZCs engage in intricate physiological processes while long-term metabolic pathways, potential accumulation, and potential immune responses remain incompletely understood. Critical questions remain regarding the long-term biological stability of AZCs *in vivo*: whether they may gradually lose their original biological properties, potentially triggering uncontrolled adverse effects or interfering with normal cellular functions and metabolism. These concerns underscore the need for thorough investigation^175^. Another challenge lies in the precision and controllability of AZCs within the body. While these cells can be engineered to target specific sites, their effectiveness can be significantly compromised in the complex physiological environment due to various influencing factors. Ensuring the accurate delivery of AZCs to target tissues and cells, as well as the precise release of therapeutic agents they carry when required, while minimizing off-target effects, represents a critical technical hurdle that requires immediate attention. Challenges exist in the preparation and standardized production of AZCs. Currently, there is a lack of consistent quality standards and standardized processes to ensure the stability of AZCs for clinical use. Inherent variability in source cells, cryogenic shock parameters, and hydrogelation conditions can critically impact the consistency of key attributes like membrane protein functionality, drug loading efficiency, size and zeta potential. Last but certainly not least, our comprehension of how AZCs interact with human cells and microorganisms is limited. The human body functions as a complex ecosystem, and the introduction of AZCs has the potential to disrupt the existing equilibrium, which may lead to unforeseeable chain reactions^176^. A comprehensive understanding of these interaction mechanisms is essential for evaluating the safety and efficacy of AZCs and facilitating the development of rational and scientifically grounded clinical applications.

Several strategies can be employed to address these challenges. Firstly, comprehensive long-term preclinical studies should be conducted to investigate metabolism, accumulation, and immune responses associated with prolonged use for ensuring safety. Secondly, enhanced *in vivo* targeting and controllability can be achieved through the utilization of intelligent drug delivery systems and real-time monitoring and regulation mechanisms to achieve precise therapeutic outcomes. Moreover, optimizing the preparation and standardizing production processes is crucial for accurately controlling each step, establishing unified quality standards, and implementing standardized procedures. This involves standardizing raw materials, implementing real-time process monitoring, and establishing strict release criteria that assess biological function—not just physical properties. Controlling such variability is fundamental to achieving reproducible safety and efficacy in clinical applications. Finally, to understand interactions between human cells and microbial communities, in-depth research employing multi-omics technology is required, together with the development of a big data platform integrating clinical sample analysis, to facilitate the formulation of evidence-based clinical application strategies.

If current limitations can be effectively addressed, AZCs will demonstrate significant potential for disease treatment applications. Standardized and quality-controlled preparation processes will enhance the accuracy and reliability of diagnostic models utilizing AZCs in terms of disease detection. Tailoring specific AZCs sensors can enable rapid and sensitive detection of disease-related molecules. In the area of disease treatment, AZCs are possible to emerge as innovative precision tools. Their capabilities as drug carriers with controlled release mechanisms hold promise for targeted drug delivery to affected areas, thereby enhancing treatment efficacy while mitigating adverse effects on healthy tissues. Furthermore, a comprehensive understanding of their *in vivo* behavior and mechanisms paves the way for targeted modifications that can imbue them with diverse therapeutic potentials. For instance, through gene editing, AZCs can be engineered to secrete specific factors, offering novel avenues for addressing challenging conditions like nervous system injuries and autoimmune disorders. Although AZCs are currently in the early stages of development and face multiple challenges, proactive efforts to address these limitations are expected to drive breakthroughs in disease diagnosis and treatment, ultimately benefiting human health.

## 6 Conclusions

In conclusion, this study defined the concept of AZCs and systematically reviewed their applications in the field of disease treatment. It was found that AZCs have demonstrated significant advantages in drug-targeted therapy and the development of cancer vaccines. At present, this type of cells has made significant therapeutic progress in multiple directions, including targeted therapy for tumors and inflammation, storage of cancer vaccines, and coordinated regulation of inflammation. However, AZCs still face several key issues, such as the lack of systematic quality evaluation standards and the insufficient assessment of long-term safety. These factors severely limit their clinical transformation. Meanwhile, AZCs also possess targeted and synergistic therapeutic capabilities. They can not only reduce the side effects of traditional drugs but also enhance therapeutic effects through reasonable design, thereby providing more treatment options for clinical patients. With the deepening of understanding of AZCs and the continuous development of related technologies, they will show broader application prospects in the future and bring better therapeutic effects to patients.

## Author contributions

Yu Sun: Writing – original draft, Writing – review and editing, Software, Methodology, Investigation, Formal analysis, Conceptualization. Jie Feng: Writing – review and editing, Validation, Visualization, Methodology, Investigation, Conceptualization. Ye Xing: Writing – review and editing, Validation, Visualization, Investigation, Formal analysis. Ping Sun: Writing – review and editing, Investigation, Methodology. Minghui Liao: Writing – review and editing, Methodology. Xiaoyan Tan: Writing – review and editing, Supervision, Validation, Visualization, Methodology. Yi Wang: Writing – review and editing, Supervision, Project administration, Funding acquisition, Conceptualization.

## Conflicts of interest

The authors declare no conflicts of interest.
